# The projections of global and regional rheumatic heart disease burden from 2020 to 2030

**DOI:** 10.3389/fcvm.2022.941917

**Published:** 2022-10-18

**Authors:** Yingying Hu, Zijia Tong, Xuewei Huang, Juan-Juan Qin, Lijin Lin, Fang Lei, Wenxin Wang, Weifang Liu, Tao Sun, Jingjing Cai, Zhi-Gang She, Hongliang Li

**Affiliations:** ^1^Department of Cardiology, Renmin Hospital of Wuhan University, Wuhan, China; ^2^Institute of Model Animal, Wuhan University, Wuhan, China; ^3^Department of Cardiology, Huanggang Central Hospital of Yangtze University, Huanggang, China; ^4^Huanggang Institute of Translational Medicine, Huanggang, China; ^5^Department of Cardiology, The Third Xiangya Hospital, Central South University, Changsha, China

**Keywords:** rheumatic heart disease, projections, disease burden, age-standardized prevalence rates, health policy

## Abstract

**Background:**

Rheumatic heart disease (RHD) remains the leading cause of preventable death and disability in children and young adults, killing an estimated 320,000 individuals worldwide yearly.

**Materials and methods:**

We utilized the Bayesian age-period cohort (BAPC) model to project the change in disease burden from 2020 to 2030 using the data from the Global Burden of Disease (GBD) Study 2019. Then we described the projected epidemiological characteristics of RHD by region, sex, and age.

**Results:**

The global age-standardized prevalence rate (ASPR) and age-standardized incidence rate (ASIR) of RHD increased from 1990 to 2019, and ASPR will increase to 559.88 per 100,000 population by 2030. The global age-standardized mortality rate (ASMR) of RHD will continue declining, while the projected death cases will increase. Furthermore, ASPR and cases of RHD-associated HF will continue rising, and there will be 2,922,840 heart failure (HF) cases in 2030 globally. Female subjects will still be the dominant population compared to male subjects, and the ASPR of RHD and the ASPR of RHD-associated HF in female subjects will continue to increase from 2020 to 2030. Young people will have the highest ASPR of RHD among all age groups globally, while the elderly will bear a greater death and HF burden.

**Conclusion:**

In the following decade, the RHD burden will remain severe. There are large variations in the trend of RHD burden by region, sex, and age. Targeted and effective strategies are needed for the management of RHD, particularly in female subjects and young people in developing regions.

## Introduction

Rheumatic heart disease (RHD), although regarded as a preventable disease, had affected 40.5 million individuals by 2019 (1), and had resulted in around 1,100,000 cases of heart failure (HF) as well as 320,000 death cases annually (2, 3). The improved living standards, access to healthcare, and the widespread use of penicillin-like drugs have alleviated the disease burden of RHD in the past decades (4). However, RHD is still a major cause of serious valvular heart disease and increases the health burden in some regions and populations (2, 3, 5, 6). In several developing countries, such as South Africa and India, RHD remains a public health priority (7, 8). Even in some developed countries, the case number of RHD in children has started to increase (5, 6). Those who suffer from RHD in childhood will face a significant burden of HF in future. These facts suggested that focused studies should be conducted to figure out the future trends of RHD and to guide more specified and effective policy-making. Some strategies, such as expanding echocardiographic screening and modifying perioperative penicillin use, could be implemented in high-risk locations.

In this study, we used the Global Burden of Disease (GBD) Study 2019 database to project the RHD-related disease burden from 2020 to 2030 and described the projected burden in prevalence, death, and heart failure by region, sex, and age. The results from this analysis would project longitudinal changes in RHD in the near future and guide the development of more targeted strategies to reduce the RHD burden.

## Materials and methods

### Study data

The annual number of indicators of RHD burden, including prevalence, incidence, mortality, and prevalence of HF by region, sex, and age from 1990 to 2019, were extracted from the Global Health Data Exchange (GHDx) query tool.^1^ The Global Burden of Diseases, Injuries, and Risk Factors Study 2019, which is supported by an ongoing multinational collaboration, comprehensively and systematically estimates 369 diseases and injuries as well as 87 behavioral, environmental, occupational, and metabolic risk factors in 204 countries and territories, 21 regions, and seven super-regions from 1990 to 2019 (1, 9). The detailed component information on data collecting and study processing used for GBD 2019 have already been thoroughly described (3). Our study complied with the Guidelines for Accurate and Transparent Health Estimates Reporting (GATHER) (Supplementary Table 1).

### Definitions

Rheumatic heart disease is a heart disease mainly affecting the heart valves, especially the mitral valve. It occurs as a cardiac involvement of acute rheumatic fever caused by the infection of group A streptococcus, frequently resulting in premature death and HF (10). More detailed diagnosis information about RHD could be acquired on the website.^2^ The sociodemographic index (SDI), collected from the GBD database, is a summary measure. It quantitates the average level in several basic dimensions of country and region achievement, such as incomes, educational attainments, and fertility rates (1, 9, 11, 12). The scale is limited to 0 to 1. 0 represents the lowest composite average level in all GBD countries, and 1 is in reverse. Based on SDI, all GBD countries are divided into five types (high, high-middle, middle, low-middle, and low levels). In addition, they are also divided into 21 GBD regions based on their geographic locations. In our research, we displayed the figure and table in the order of decreasing average SDI values for the 21 GBD regions. Death data were obtained from the different national vital registration databases, verbal autopsies, or household mortality survey records. The Cause of Death Ensemble model (CODEm) was used to estimate the cause-specific mortality of RHD for the region, sex, age, and year. The cause of death was coded in the International Classification of Diseases (ICD) system and then mapped to the RHD. The ICD-10 codes matched to RHD are I01-I01.9, I02.0, and I05-I09.9.

### Statistical analysis

#### Age-standardized rate and estimated annual percentage change

Specific indicators reported by region, sex, age, and year measuring the burden of RHD in our research involved prevalence, incidence, mortality, and prevalence of HF. The RHD-associated burden was statistically estimated and modeled, utilizing an integrated and multi-parameter approach. The count, age-standardized rate (ASR) per 100,000 population, and 95% UI extracted from the database were used to quantify the burden of RHD. ASR was the rate per 100,000 people following a standardized global age structure, which could be used to exclude the effects of aging as much as possible (13).

Temporal trends of ASR from 2020 to 2030 were quantified by the estimated annual percentage change (EAPC) using least squares linear regression. The EAPC is a summative and widely used measurement for ASR trends over specified intervals (14). If the EAPC estimation and its lower limit of 95% CI were both positive, the ASR was considered to be in an increasing trend. Conversely, if the EAPC estimation and its upper limit of 95% CI were both negative, the ASR was considered to be in a downward trend. Otherwise, the ASR was deemed to be stable.

#### Forecasting model development

We used the GBD data from 1990 to 2019 to project the disease burden from 2020 to 2030. First, we collected the prevalence, death, and HF cases of RHD for all age groups (in 5-year intervals) at the global and regional levels from 1990 to 2019. Second, according to the formula, the cases of prevalence (or death or HF) for all age groups in a certain year/corresponding rate for all age groups in the same year, we restored the corresponding annual total populations. And then, the BAPC model was used to project the disease burden from 2020 to 2030.

The superior predictive performance of the BAPC model has been verified (15, 16). BAPC model assumed a similar effect of age, period, and cohort adjacent in time. And all unknown parameters were regarded as random with appropriate prior distributions in the BAPC model. The Bayesian inference used the second-order random walk for smoothing priors of age, period, and cohort effects. The prior knowledge combined with observed data was used to derive a posterior distribution (17). The integrated nested Laplace approximations were used with the BAPC model to approximate the marginal posterior distributions, avoiding mixing and convergence issues introduced by Markov chain Monte Carlo sampling techniques traditionally used in the Bayesian approach. We conducted the BAPC analysis using R-package BAPC (version 0.0.34).

The mean absolute percentage error (MAPE) was applied to calculate the projection deviance to evaluate model performance (18). The data on prevalence, death, and heart failure of RHD at the global level and in different SDI regions from 1990 to 2019 were retrieved and split into two periods: 1990–2013 and 2014–2019. Data from 1990 to 2013 were used to project data from 2014 to 2019 by the BAPC model, and projected data were compared with the true values. Supplementary Table 2 shows the MAPE results at the global level and in the five SDI regions.

The R program (Version 4.1.0, R Core Team) was used to perform the statistical analysis. All graphs were drafted by GraphPad Prism (Version 8.0.2) and R program.

## Results

### The changes in rheumatic heart disease burden from 2020 to 2030 at the global level

**FIGURE 1:**
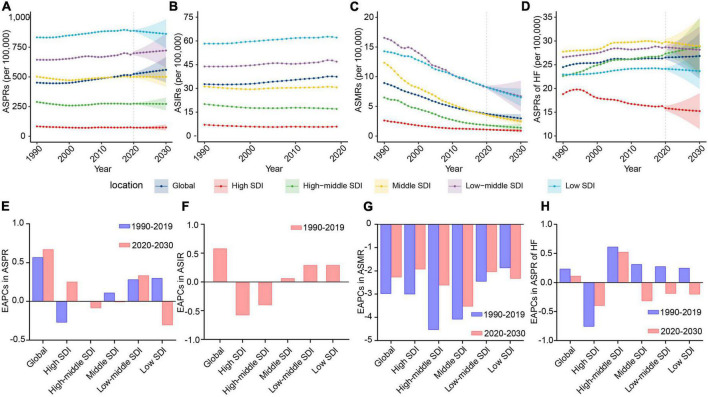
The change trends of the RHD-related disease burden at the global level and in different SDI regions. **(A)** The ASPRs from 1990 to 2030. **(B)** The ASIRs from 1990 to 2019. **(C)** The ASMRs from 1990 to 2030. **(D)** The ASPRs of HF from 1990 to 2030. **(E)** The EAPCs in ASPR between 1990–2019 and 2020–2030. **(F)** The EAPCs in ASIR between 1990 and 2019. **(G)** The EAPCs in ASMR between 1990–2019 and 2020–2030. **(H)** The EAPCs in ASPRs of HF between 1990–2019 and 2020–2030. RHD, rheumatic heart disease; SDI, sociodemographic index; ASPRs, age-standardized prevalence rates; ASIRs, age-standardized incidence rates; ASMRs, age-standardized mortality rates; HF, heart failure; EAPCs, estimated annual percentage changes.

There are rising trends in ASPR and ASIR of RHD from 1990 to 2019 at the global level (Figures 1A,B). Predictively, the ASPR will still maintain an upward trend, from 523.86 per 100,000 (95%CI, 511.92–535.81) to 559.88 per 100,000 (95%CI, 452.19–667.58), in the following decade (Figure 1E and Table 1). We could not project the future changes in incidence burden due to a lack of data (Figures 1B,F). Although RHD-related death cases will continue rising (Supplementary Table 3), the ASMR will decrease from 2020 to 2030 (Figures 1C,G and Supplementary Table 5). Heart failure is a major complication and reason for hospitalization for individuals with RHD. From 2020 to 2030, ASPR and cases of RHD-associated HF will continue increasing (Figures 1D,H and Supplementary Table 6). The ASPR and cases of HF will be 26.84 per 100,000 (95%CI, 22.09–31.59) and 2,922,840 (95%CI, 2,752,383–3,093,297) by 2030, respectively (Supplementary Tables 3, 6).

**TABLE 1:** The RHD-related ASPRs in 1990, 2020, and 2030, and their temporal trends from 1990 to 2019 and from 2020 to 2030.

	1990	2020	2030	1990–2019	2020–2030
	
Characteristics	ASPRs per 100, 000 (95% UI)	ASPRs per 100, 000 (95% CI)	ASPRs per 100, 000 (95% CI)	EAPC of ASPRs (95% CI)	EAPC of ASPRs (95% CI)
Global	451.56 (363.35,552.54)	523.86 (511.92,535.81)	559.88 (452.19,667.58)	0.57 (0.50,0.63)	0.67 (0.66,0.68)
**Sex**					
Female	501.56 (405.50,609.67)	582.20 (569.08,595.32)	631.51 (510.17,752.85)	0.55 (0.48,0.63)	0.82 (0.81,0.83)
Male	400.46 (321.44,492.36)	465.35 (454.84,475.86)	487.20 (394.83,579.58)	0.59 (0.53,0.65)	0.46 (0.45,0.47)
**SDI**					
High SDI	82.93 (71.82,93.74)	72.51 (69.45,75.58)	74.38 (49.24,99.52)	−0.27 (−0.41, −0.13)	0.25 (0.21,0.29)
High-middle SDI	289.23 (246.15,342.21)	274.56 (269.00,280.12)	272.22 (217.30,327.14)	0.00 (−0.13,0.13)	−0.09 (−0.09, −0.08)
Middle SDI	501.87 (396.81,624.96)	500.87 (491.10,510.63)	500.46 (414.40,586.52)	0.11 (0.03,0.19)	−0.01 (−0.01, −0.01)
Low-middle SDI	644.37 (503.97,805.80)	699.12 (681.18,717.06)	722.54 (578.21,866.86)	0.28 (0.25,0.31)	0.33 (0.33,0.34)
Low SDI	832.91 (648.55,1039.27)	889.00 (879.72,898.29)	862.32 (739.79,984.84)	0.30 (0.28,0.32)	−0.30 (−0.31, −0.30)
**Regions**					
High-income Asia Pacific	49.45 (41.49,57.17)	35.01 (33.64,36.38)	35.75 (22.83,48.66)	−1.58 (−1.69, −1.46)	0.20 (0.20,0.21)
High-income North America	123.49 (104.25,142.40)	112.41 (107.29,117.54)	114.30 (80.36,148.24)	0.02 (−0.17,0.22)	0.16 (0.14,0.19)
Western Europe	55.18 (47.96,63.00)	40.70 (39.75,41.65)	40.82 (31.92,49.71)	−1.27 (−1.34, −1.20)	0.02 (0.01,0.03)
Australasia	61.01 (51.93,70.06)	51.32 (50.15,52.49)	56.75 (43.77,69.72)	−0.76 (−0.92, −0.59)	1.01 (0.99,1.03)
Eastern Europe	215.61 (190.56,244.46)	133.72 (129.43,138.01)	137.61 (95.11,180.10)	−1.62 (−1.83, −1.41)	0.28 (0.24,0.32)
Central Europe	144.04 (129.59,160.06)	93.45 (90.35,96.56)	76.50 (55.50,97.49)	−1.29 (−1.40, −1.17)	−1.98 (−1.99, −1.97)
Southern Latin America	469.70 (374.66,582.67)	490.51 (484.78,496.23)	513.92 (437.16,590.68)	0.15 (0.12,0.18)	0.47 (0.47,0.47)
East Asia	441.29 (349.90,549.84)	388.17 (377.45,398.89)	394.11 (301.95,486.27)	−0.22 (−0.40, −0.04)	0.15 (0.15,0.15)
Central Asia	590.89 (470.83,729.78)	628.40 (617.66,639.14)	626.48 (522.89,730.07)	0.19 (0.17,0.21)	−0.03 (−0.04, −0.02)
North Africa and Middle East	368.81 (293.17,455.91)	394.27 (388.55,400.00)	415.03 (347.83,482.23)	0.22 (0.16,0.27)	0.51 (0.51,0.52)
Southeast Asia	274.88 (225.94,333.61)	287.17 (282.83,291.50)	300.84 (249.08,352.60)	0.15 (0.11,0.19)	0.47 (0.46,0.48)
Southern Sub-Saharan Africa	1071.74 (835.28,1350.28)	1091.38 (1079.84,1102.92)	1093.31 (939.03,1247.59)	0.04 (0.03,0.06)	0.02 (0.02,0.02)
Tropical Latin America	899.05 (699.28,1119.15)	917.52 (908.95,926.09)	919.65 (799.92,1039.39)	0.08 (0.07,0.09)	0.02 (0.02,0.02)
Andean Latin America	793.87 (615.64,992.80)	813.21 (804.31,822.12)	815.35 (700.40,930.29)	0.09 (0.06,0.11)	0.03 (0.02,0.03)
Caribbean	747.85 (581.94,932.43)	788.04 (779.19,796.88)	795.44 (683.47,907.41)	0.18 (0.18,0.18)	0.09 (0.09,0.10)
Central Latin America	358.19 (287.12,435.28)	355.91 (352.05,359.78)	370.99 (316.80,425.17)	−0.07 (−0.14,0.00)	0.42 (0.41,0.42)
South Asia	623.88 (481.49,781.45)	662.22 (631.56,692.88)	666.98 (500.17,833.79)	0.26 (0.21,0.31)	0.08 (0.07,0.09)
Central Sub-Saharan Africa	1184.76 (910.15,1512.89)	1196.93 (1184.56,1209.30)	1212.34 (1047.07,1377.60)	−0.03 (−0.05, −0.01)	0.13 (0.12,0.13)
Oceania	543.08 (430.73,690.95)	587.12 (577.08,597.16)	588.23 (470.59,705.86)	0.34 (0.27,0.41)	0.02 (0.02,0.02)
Western Sub-Saharan Africa	794.99 (618.33,1001.81)	839.98 (831.77,848.19)	832.77 (721.05,944.48)	0.22 (0.19,0.24)	−0.09 (−0.09, −0.08)
Eastern Sub-Saharan Africa	1093.85 (849.57,1370.07)	1179.01 (1166.38,1191.64)	1187.93 (1015.00,1360.87)	0.27 (0.25,0.28)	0.08 (0.07,0.08)

RHD, rheumatic heart disease; ASPRs, age-standardized prevalence rates; EAPC, estimated annual percentage change; UI, uncertainty interval; CI, confidence interval; SDI, sociodemographic index.

### The changes in rheumatic heart disease burden from 2020 to 2030 in different sociodemographic index regions

To assist in tailoring targeted preventive strategies for different areas, we further analyzed the RHD burden in five SDI regions. The results showed that the ASPR of RHD in low-middle and high SDI regions will increase from 2020 to 2030, while other SDI regions will experience a steady and significant decline (Figures 1A,E and Table 1). By 2030, the highest ASPR was expected to be observed in the low SDI region among five SDI regions; on the contrary, the lowest ASPR will be in the high SDI region (Figure 1A). Therefore, the RHD-related prevalence rate in developing regions will still be intense; the uptrend in developed regions will also merit concerns. Due to a lack of data, we could not project the future changes in incidence burden. The trend in ASIR of RHD was consistent with the trend of ASPR in the past (Figures 1B,F and Supplementary Table 4). Regarding the mortality burden, ASMR will decline in all SDI regions in the forecast period (Figures 1C,G). At the end of the study period, the highest ASMR will be in the low-middle SDI region, followed by the low SDI region (Figure 1C). When considering HF, the major complication of RHD, the ASPR of HF was projected to increase dramatically in the high-middle SDI region in the following decade, reaching 28.82 (95% CI, 22.72–34.93) per 100,000 individuals in 2030 (Figures 1D,H and Supplementary Table 6). Therefore, the ASPR of HF in the high-middle SDI region will tend to surpass that in the middle SDI area and rank first (Figure 1D). In contrast, the ASPR of HF in other SDI regions will experience a steady decline from 2020 to 2030 (Figures 1D,H). Moreover, the ASPR of HF will decline much faster in the high SDI region than in middle, low-middle, and low SDI areas (Figure 1H).

### The changes in rheumatic heart disease burden from 2020 to 2030 in different geographic regions

**FIGURE 2:**
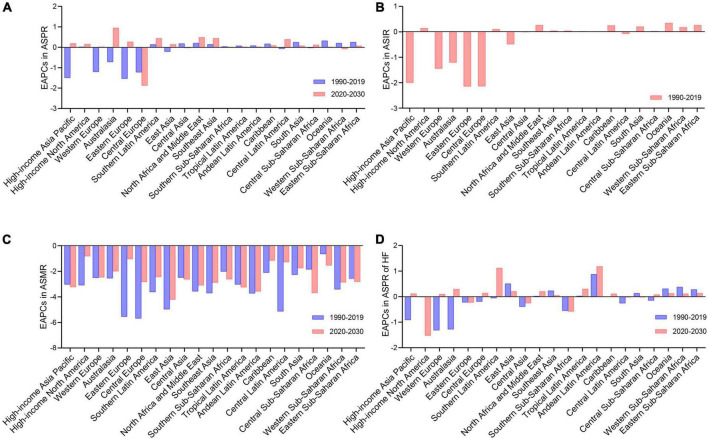
The change trends of the RHD-related disease burden in 21 GBD regions. **(A)** The EAPCs in ASPR between 1990–2019 and 2020–2030. **(B)** The EAPCs in ASIR between 1990 and 2019. **(C)** The EAPCs in ASMR between 1990–2019 and 2020–2030. **(D)** The EAPCs in ASPR of HF between 1990–2019 and 2020–2030. RHD, rheumatic heart disease; GBD, global burden of disease; EAPCs, estimated annual percentage changes; ASPR, age-standardized prevalence rate; ASIR, age-standardized incidence rate; ASMR, age-standardized mortality rate; HF, heart failure.

Based on the epidemiologic data in 21 GBD regions, the ASPR of RHD will increase in 18 GBD regions from 2020 to 2030, with the fastest increase rate in Australasia (EAPC = 1.01 [95% CI, 0.99–1.03]) (Figure 2A and Table 1). By 2030, the highest ASPR will be in Central, Eastern, and Southern Sub-Saharan Africa among 21 GBD regions (Supplementary Figure 1A). The RHD-related ASIR followed a similar trend as the ASPR in the past three decades (Figure 2B and Supplementary Figure 1B). From 2020 to 2030, all GBD regions were estimated to experience declines in ASMR, while the slowest decrease rate will be in high-income North America (EAPC = −0.82 [95% CI, −0.84 to −0.81]), followed by Eastern Europe, Caribbean and Central Latin America (EAPC: −1.05 to −1.29) (Figure 2C and Supplementary Table 5). By 2030, the highest ASMR was observed in Oceania and South Asia (Supplementary Figure 1C). Regarding the ASPR of RHD-associated HF, rising trends were projected to be observed in 15 GBD regions (Figure 2D and Supplementary Table 6). By 2030, the top ASPR of HF will be in East Asia, Oceania, and Western Sub-Saharan Africa, while Eastern Sub-Saharan Africa, Tropical Latin America, and Central Sub-Saharan Africa will have the lowest (Supplementary Figure 1D).

### The changes in rheumatic heart disease burden from 2020 to 2030 in different sexes

**FIGURE 3:**
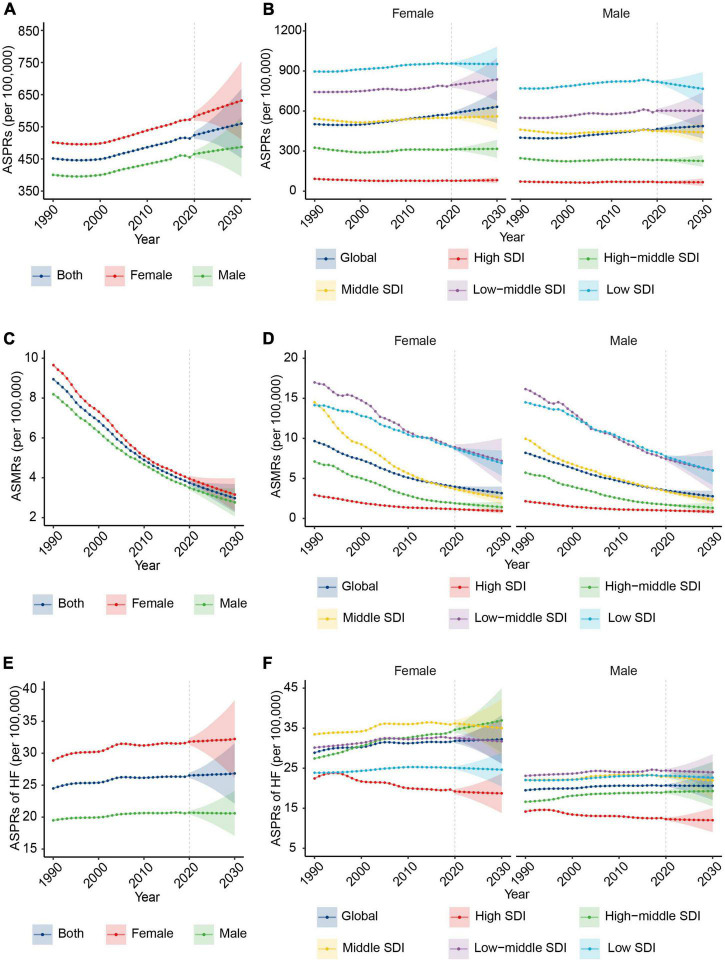
The change trends of the RHD-related disease burden by sex from 1990 to 2030. **(A)** The ASPRs from 1990 to 2030 at the global level by sex. **(B)** The ASPRs at the global level and in different SDI regions by female (the left) and male (the right) subjects. **(C)** The ASMRs from 1990 to 2030 at the global level by sex. **(D)** The ASMRs from 1990 to 2030 at the global level and in different SDI regions by female (the left) and male (the right) subjects. **(E)** The ASPRs of HF from 1990 to 2030 at the global level by sex. **(F)** The ASPRs of HF from 1990 to 2030 at the global level and in different SDI regions by female (the left) and male (the right) subjects. RHD, rheumatic heart disease; ASPRs, age-standardized prevalence rates; SDI, sociodemographic index; ASMRs, age-standardized mortality rates; HF, heart failure.

To identify the sex group that needs focused attention regarding RHD burden, we further analyzed the sex differences in RHD-related ASPR, ASMR, and ASPR of HF at the global level and in different SDI regions. Globally, RHD-related prevalence, death, and HF burden will remain higher in female subjects than in male subjects by 2030 (Figures 3A,C,E), and the gap between female and male subjects will further increase from 2020 to 2030 (Supplementary Figures 2A–C). The ASPR and ASMR showed a similar trend in female and male subjects from 2020 to 2030 (Supplementary Figures 3A,B). Regarding the ASPR of HF, female subjects will sustain a rising trend in the following decade, while male subjects will have a downward trend. The increase in the ASPR of HF in female subjects will result in a growth of HF ASPR global wide (Figure 1H and Supplementary Figure 3C).

At the SDI levels, there will be a rising trend in ASPR in female subjects in the majority of SDI regions, while the declining trend will be in male subjects (Figure 3B and Supplementary Figure 3A). The descending rate of ASMR in female subjects will be slower than in male subjects in the low SDI region from 2020 to 2030 (Figure 3D and Supplementary Figure 3B). In 2030, the region with the highest ASPR of HF in female subjects will shift from the middle region to the high-middle SDI region, and the ASPR of HF in the high-middle SDI region will keep an upward trend (Figure 3F).

### The changes in rheumatic heart disease burden from 2020 to 2030 in different age groups

**FIGURE 4:**
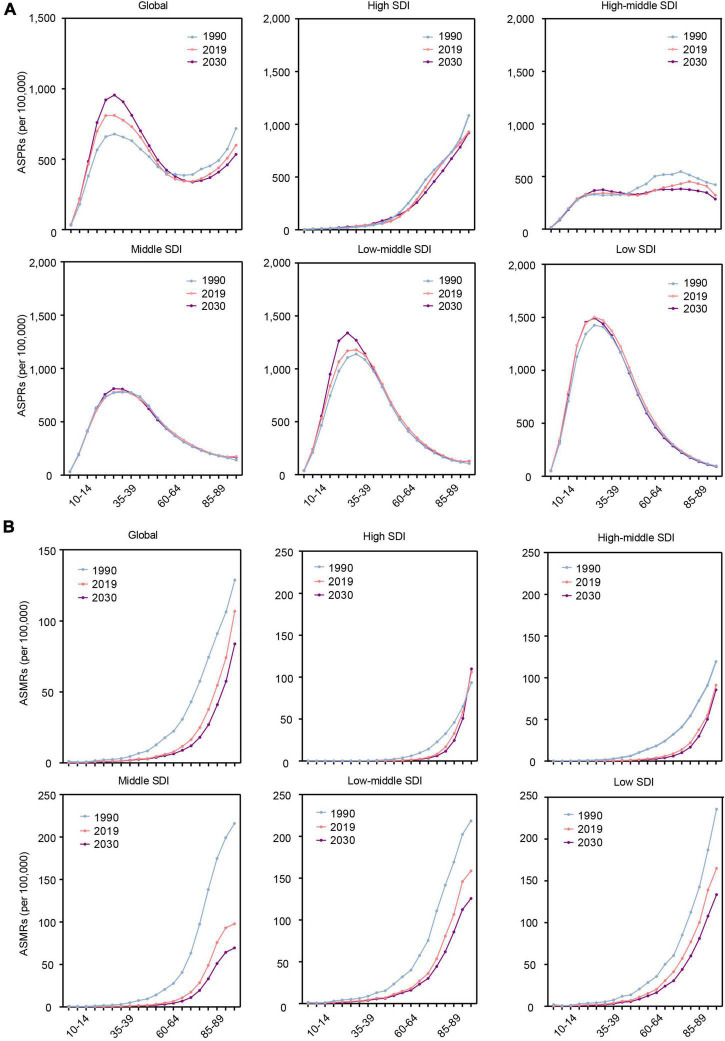
The change trends of the RHD-related disease burden at the global level and in different SDI regions by age in 1990, 2019, and 2030. **(A)** The ASPRs. **(B)** The ASMRs. RHD, rheumatic heart disease; SDI, sociodemographic index; ASPRs, age-standardized prevalence rates; ASMRs, age-standardized mortality rates.

We further estimated the RHD-related burden in age groups globally and in different SDI regions. Globally, the ASPR of RHD will increase in the young population (10–54 age group) by 2030 (Figure 4A). The ASMR of RHD will decrease in all age groups (Figure 4B). The ASPR of RHD-related HF will increase in the elderly (55–84 age group) (Supplementary Figure 4).

In different SDI regions, the highest ASPR of RHD will be in the 20–34 age group among all age groups, and it will be mainly concentrated in low and low-middle SDI regions. In the high SDI region, the highest ASPR of RHD will be observed in the elderly among all age groups (Figure 4A). The ASMR of RHD will increase with age in all SDI regions. Simultaneously, the senior population will account for the majority population for RHD-related death from 2020 to 2030 (Figure 4B). For ASPR of RHD-related HF, in low and low-middle regions, the highest ASPR of HF will be in the 65–74 age group among all age groups, while in the high SDI region, it will be in the 85 plus age group (Supplementary Figure 4).

## Discussion

Rheumatic heart disease remains the leading cause of severe valvular heart disease, with significant regional disparities. Our study is the first to project the RHD burden at global and regional levels from 2020 to 2030. According to our analysis, the ASPR of RHD and the ASPR of RHD-related HF will keep increasing globally from 2020 to 2030, although the ASMR of RHD will decline. Therefore, the global burden of RHD has not been sufficiently controlled. It is urgent to implement effective and precise strategies to control RHD and the related disease burden based on demographic characteristics.

Rheumatic heart disease results in immense medical and economic burdens (19, 20). The cost of RHD treatment was the highest among all cardiovascular diseases (averaged US $ 4710.78) (21). According to the projection of our research, the prevalence cases of RHD will exceed 48 million in 2030, continuously leading to a substantial economic burden in the near future. Premature mortality from RHD shortens life expectations significantly. Meanwhile, RHD patients with HF are more susceptible to infection, enhancing the risk of developing infective endocarditis. Moreover, increased antibiotic use in these patients raises the chance of enriching drug-resistant bacteria. Simultaneously, clinical management becomes more challenging when RHD progresses rapidly. Therefore, more attention should be paid to preventing and treating RHD and related diseases to alleviate the huge burden.

Our study indicates that the ASPR in the low SDI region will decrease from 2020 to 2030. Over the past three decades, poor sanitation, overcrowding, and limited access to healthcare have resulted in the highest prevalence and a rising trend in the low SDI area (19). Accordingly, extensive interventions to improve the efficacy of the prevention and management of RHD have been implemented in developing regions (22–26). In 2015, seven priority actions were developed in Africa. These actions include creating disease registers, ensuring the supply of benzathine penicillin, improving access to reproductive health services for female subjects, decentralizing technical expertise and technology, establishing centers for essential cardiac surgery, initiating national multi-sectoral RHD programmers, fostering international partnerships, and have been implemented in a large portion of African countries to eliminate RHD since then (26). It has been reported that RHD in African nations, such as Cameroon, Ethiopia, and Uganda, had significantly lower prevalence rates after 2015 than those counted before 2015 (27). These results indicate the success of the positive prevention in reducing the prevalence of RHD. We here project that the prevalence in the low SDI area will decrease from 2020 to 2030, further remarking the necessity of preventive efforts. Notably, our results suggest that the highest ASPR will last in the low SDI region by 2030. Therefore, developing regions should further strengthen prevention and management strategies to decrease the prevalence of RHD.

Another key finding in this study is the downward trend in ASPR of RHD in the high SDI area over the past three decades will be reversed in the following decade. In the past three decades, the prevalence of RHD in the high SDI region has decreased mainly due to improvements in the social environment and extensive use of penicillin. However, diminishing alerts of physicians in the diagnosis of RHD, lack of awareness about RHD prevention and reduced compliance with penicillin treatment have also emerged (28, 29). These issues have contributed to a rise in the prevalence of RHD in developed regions in the recent decade (30–35). Our study projects that the prevalence of RHD in developed regions will increase over the next decade if effective interventions are not fulfilled in time. In this regard, indigenous populations remain the primary concern (30, 36–38), particularly for indigenous Australians, as the highest incidence of RHD in the world was found in this population (39). This situation could be associated with the following facts. First, there is no uniform consensus in Australasia on how to improve the primary prevention of RHD (40). Second, there are disparities in diverse practices among various indigenous populations, restricting their access to healthcare, housing, and education (41–44). Third, previous health warning campaigns have triggered resistance in indigenous populations due to communication obstacles (44–46). Fourth, the ethnic background and hereditary may facilitate the development of RHD (47). These adverse rising trends in the high SDI area might constitute a major obstacle for RHD prevention and management. Therefore, effective measures should be fulfilled as soon as possible to reverse this negative tendency even in developed regions.

However, our study shows a decline in ASMR, with huge variation across all SDI regions from 2020 to 2030. The ratio of low SDI region to the high SDI region will be more than seven times. This inequality is primarily attributable to regional disparities in educational achievement and access to healthcare (48). Access to cardiac surgical therapy in developing regions is severely restricted due to the scarcity of heart surgery equipment and healthcare workforces (49–51), resulting in a much higher mortality rate in developing regions than in developed regions. It is known that the severity of valve lesions is the strongest predictor of mortality (52). The capacity to diagnose valve lesions in the early stage significantly varies and results in regional disparities in mortality rates. In this regard, echocardiography has been a cornerstone in screening programs to evaluate the prevalence of RHD and the severity of valve lesions according to multiple guidelines (53), since echocardiography could identify individuals with RHD-related valvular lesions early, confirm the severity of lesions and evaluate the prognosis to achieve early control and management of RHD (54), even in patients with RHD without overt clinical findings (subclinical carditis) (55). Therefore, significant human and material resources, additional capital investment, and extensive government efforts are required in developing regions to further decrease mortality (56, 57).

This study has several limitations. First, due to the nature of the GBD study, the data quality varies greatly between countries and regions. Our study utilized various modeling processes to compensate for this limitation and presents metrics with 95% UIs. Second, the RHD burden might be underestimated due to insufficient RHD screening in many regions, especially in low and low-middle SDI areas. Third, our study is conducted at global and regional levels without further probing the heterogeneity of endemic and non-endemic regions within countries. Fourth, the future changes in incidence burden could not be projected due to a lack of data.

## Conclusion

Rheumatic heart disease burden will still be serious in the next decade, with significant regional variability. Except for focusing on low and low-middle SDI regions with the highest prevalence rate and death burden, it is still necessary to control the rising trend in developed countries to avoid the resurgence of the diseases. Female subjects need to be particularly cared for in controlling RHD and appropriate policies and the inclination of medical resources are needed. The highest prevalence is concentrated in younger people among all age groups, while the elderly will have the highest burden of death and heart failure. To diminish the disease burden of RHD, precise and targeted strategies to control the burden of RHD need to be developed based on regional and population characteristics.

## Data availability statement

Publicly available datasets were analyzed in this study. This data can be found here: http://ghdx.healthdata.org/gbd-results-tool.

## Author contributions

YH and ZT designed the study, collected and analyzed data, and wrote the manuscript. XH, J-JQ, and LL collected and reviewed data and contributed to data analysis. FL, WW, WL, TS, and JC revised the manuscript and provided valuable suggestions for study design and data analysis. Z-GS and HL designed the project, edited the manuscript, and supervised the study. All authors have approved the final version of this article.

## Footnotes

1
http://ghdx.healthdata.org/gbd-results-tool

2
http://www.healthdata.org/results/gbd_summaries/2019

## Abbreviations

ASIR: age-standardized prevalence rate
ASMR: age-standardized mortality rate
ASPR: age-standardized prevalence rate
ASR: age-standardized rate
BAPC: Bayesian age-period cohort
CI: confidence interval
CODEm: Cause of Death Ensemble model
EAPC: estimated annual percentage change
GBD: Global Burden of Disease
GHDx: Global Health Data Exchange
HF: heart failure
ICD: International Classification of Diseases
MAPE: mean absolute percentage error
RHD: rheumatic heart disease
SDI: sociodemographic index
UI: uncertainty interval.

## References

[1] Roth GA Mensah GA Johnson CO Addolorato G Ammirati E Baddour LM Global burden of cardiovascular diseases and risk factors, 1990-2019: update from the GBD 2019 Study. *J Am Coll Cardiol.* (2020) 76:2982–3021. 10.1016/j.jacc.2020.11.010 33309175 PMC7755038

[2] Watkins DA Beaton AZ Carapetis JR Karthikeyan G Mayosi BM Wyber R Rheumatic heart disease worldwide: JACC scientific expert panel. *J Am Coll Cardiol.* (2018) 72:1397–416. 10.1016/j.jacc.2018.06.063 30213333

[3] Watkins DA Johnson CO Colquhoun SM Karthikeyan G Beaton A Bukhman G Global, regional, and national burden of rheumatic heart disease, 1990-2015. *N Engl J Med.* (2017) 377:713–22. 10.1056/NEJMoa1603693 28834488

[4] Carapetis JR Beaton A Cunningham MW Guilherme L Karthikeyan G Mayosi BM Acute rheumatic fever and rheumatic heart disease. *Nat Rev Dis Primers.* (2016) 2:15084. 10.1038/nrdp.2015.84 27188830 PMC5810582

[5] de Loizaga SR Beaton AZ. Rheumatic fever and rheumatic heart disease in the United States. *Pediatr Ann.* (2021) 50:e98–104. 10.3928/19382359-20210221-01 34038651

[6] Wyber R Wade V Anderson A Schreiber Y Saginur R Brown A Rheumatic heart disease in indigenous young peoples. *Lancet Child Adolesc Health.* (2021) 5:437–46. 10.1016/S2352-4642(20)30308-4 33705693

[7] Muhamed B Mutithu D Aremu O Zuhlke L Sliwa K. Rheumatic fever and rheumatic heart disease: facts and research progress in Africa. *Int J Cardiol.* (2019) 295:48–55. 31405583 10.1016/j.ijcard.2019.07.079

[8] Karthikeyan G. Rheumatic heart disease in India: declining, but not fast enough. *Natl Med J India.* (2017) 30:247–8. 10.4103/0970-258X.234389 29916422

[9] GBD 2019 Diseases and Injuries Collaborators. Global burden of 369 diseases and injuries in 204 countries and territories, 1990-2019: a systematic analysis for the global burden of disease study 2019. *Lancet.* (2020) 396:1204–22. 10.1016/S0140-6736(20)30925-9 33069326 PMC7567026

[10] Karthikeyan G Guilherme L. Acute rheumatic fever. *Lancet.* (2018) 392:161–74. 10.1016/S0140-6736(18)30999-1 30025809

[11] Mensah GA Roth GA Fuster V. The global burden of cardiovascular diseases and risk factors: 2020 and beyond. *J Am Coll Cardiol.* (2019) 74:2529–32. 10.1016/j.jacc.2019.10.009 31727292

[12] GBD 2019 Risk Factors Collaborators. Global burden of 87 risk factors in 204 countries and territories, 1990-2019: a systematic analysis for the global burden of disease study 2019. *Lancet.* (2020) 396:1223–49. 10.1016/S0140-6736(20)30752-2 33069327 PMC7566194

[13] Cao G Liu J Liu M. Global, regional, and national incidence and mortality of neonatal preterm birth, 1990-2019. *JAMA Pediatr.* (2022) 176:787–96. 10.1001/jamapediatrics.2022.1622 35639401 PMC9157382

[14] Smith-Bindman R Kwan ML Marlow EC Theis MK Bolch W Cheng SY Trends in use of medical imaging in US health care systems and in Ontario, Canada, 2000-2016. *JAMA.* (2019) 322:843–56. 10.1001/jama.2019.11456 31479136 PMC6724186

[15] Liu Z Xu K Jiang Y Cai N Fan J Mao X Global trend of aetiology-based primary liver cancer incidence from 1990 to 2030: a modelling study. *Int J Epidemiol.* (2021) 50:128–42. 10.1093/ije/dyaa196 33349860

[16] Du Z Chen W Xia Q Shi O Chen Q. Trends and projections of kidney cancer incidence at the global and national levels, 1990-2030: a bayesian age-period-cohort modeling study. *Biomark Res.* (2020) 8:16. 10.1186/s40364-020-00195-3 32435498 PMC7222434

[17] Riebler A Held L. Projecting the future burden of cancer: bayesian age-period-cohort analysis with integrated nested laplace approximations. *Biom J.* (2017) 59:531–49. 10.1002/bimj.201500263 28139001

[18] Arik SO Shor J Sinha R Yoon J Ledsam JR Le LT A prospective evaluation of ai-augmented epidemiology to forecast Covid-19 in the USA and Japan. *NPJ Digit Med.* (2021) 4:146. 10.1038/s41746-021-00511-7 34625656 PMC8501040

[19] Coffey S Roberts-Thomson R Brown A Carapetis J Chen M Enriquez-Sarano M Global epidemiology of valvular heart disease. *Nat Rev Cardiol.* (2021) 18:853–64. 10.1038/s41569-021-00570-z 34172950

[20] Marijon E Mocumbi A Narayanan K Jouven X Celermajer DS. Persisting burden and challenges of rheumatic heart disease. *Eur Heart J.* (2021) 42:3338–48. 10.1093/eurheartj/ehab407 34263296

[21] Kumar A Siddharth V Singh SI Narang R. Cost analysis of treating cardiovascular diseases in a super-specialty hospital. *PLoS One.* (2022) 17:e0262190. 10.1371/journal.pone.0262190 34986193 PMC8730466

[22] Beaton A Sable C. Health policy: reducing rheumatic heart disease in Africa – time for action. *Nat Rev Cardiol.* (2016) 13:190–1. 10.1038/nrcardio.2016.28 26935032

[23] Enumah ZO Boateng P Bolman RM Beyersdorf F Zühlke L Musoni M Societies of futures past: examining the history and potential of international society collaborations in addressing the burden of rheumatic heart disease in the developing world. *Front Cardiovasc Med.* (2021) 8:740745. 10.3389/fcvm.2021.740745 34796211 PMC8592898

[24] Coates MM Sliwa K Watkins DA Zühlke L Perel P Berteletti F An investment case for the prevention and management of rheumatic heart disease in the African Union 2021-30: a modelling study. *Lancet Glob Health.* (2021) 9:e957–66. 10.1016/s2214-109x(21)00199-6 33984296 PMC9087136

[25] Mayosi BM Gamra H Dangou JM Kasonde J. Rheumatic heart disease in Africa: the mosi-o-tunya call to action. *Lancet Glob Health.* (2014) 2:e438–9. 10.1016/s2214-109x(14)70234-7 25103507

[26] Watkins D Zuhlke L Engel M Daniels R Francis V Shaboodien G Seven key actions to eradicate rheumatic heart disease in Africa: the addis ababa communiqué. *Cardiovasc J Afr.* (2016) 27:184–7. 10.5830/cvja-2015-090 26815006 PMC5125265

[27] Muhamed B Mutithu D Aremu O Zühlke L Sliwa K. Rheumatic fever and rheumatic heart disease: facts and research progress in Africa. *Int J Cardiol.* (2019) 295:48–55. 10.1016/j.ijcard.2019.07.079 31405583

[28] Gewitz MH Baltimore RS Tani LY Sable CA Shulman ST Carapetis J Revision of the Jones criteria for the diagnosis of acute rheumatic fever in the era of doppler echocardiography: a scientific statement from the American Heart Association. *Circulation.* (2015) 131:1806–18. 10.1161/cir.0000000000000205 25908771

[29] Di Muzio I d’Angelo DM Di Battista C Lapergola G Zenobi I Marzetti V Pediatrician’s approach to diagnosis and management of group a streptococcal pharyngitis. *Eur J Clin Microbiol Infect Dis.* (2020) 39:1103–7. 10.1007/s10096-020-03821-y 31984431

[30] de Loizaga SR Arthur L Arya B Beckman B Belay W Brokamp C Rheumatic heart disease in the United States: forgotten but not gone: results of a 10 year multicenter review. *J Am Heart Assoc.* (2021) 10:e020992. 10.1161/jaha.120.020992 34348475 PMC8475057

[31] Sato S Uejima Y Suganuma E Takano T Kawano YA. Retrospective study: acute rheumatic fever and post-streptococcal reactive arthritis in Japan. *Allergol Int.* (2017) 66:617–20. 10.1016/j.alit.2017.04.001 28442182

[32] Pastore S De Cunto A Benettoni A Berton E Taddio A Lepore L. The Resurgence of rheumatic fever in a developed country area: the role of echocardiography. *Rheumatology (Oxford).* (2011) 50:396–400. 10.1093/rheumatology/keq290 21047802

[33] Licciardi F Scaioli G Mulatero R Marolda A Delle Piane M Martino S Epidemiologic impact of the new guidelines for the diagnosis of acute rheumatic fever. *J Pediatr.* (2018) 198:25–8.e1. 10.1016/j.jpeds.2018.02.024 29605389

[34] Alberio AMQ Pieroni F Di Gangi A Cappelli S Bini G Abu-Rumeileh S Toward the knowledge of the epidemiological impact of acute rheumatic fever in Italy. *Front Pediatr.* (2021) 9:746505. 10.3389/fped.2021.746505 34976887 PMC8714836

[35] Kočevar U Toplak N Kosmač B Kopač L Vesel S Krajnc N Acute rheumatic fever outbreak in southern central European country. *Eur J Pediatr.* (2017) 176:23–9. 10.1007/s00431-016-2801-z 27815733

[36] Veasy LG Tani LY Hill HR. Persistence of acute rheumatic fever in the intermountain area of the United States. *J Pediatr.* (1994) 124:9–16. 10.1016/s0022-3476(94)70247-0 7802743

[37] Lawrence JG Carapetis JR Griffiths K Edwards K Condon JR. Acute rheumatic fever and rheumatic heart disease: incidence and progression in the northern territory of Australia, 1997 to 2010. *Circulation.* (2013) 128:492–501. 10.1161/circulationaha.113.001477 23794730

[38] Baker MG Gurney J Oliver J Moreland NJ Williamson DA Pierse N Risk factors for acute rheumatic fever: literature review and protocol for a case-control study in New Zealand. *Int J Environ Res Public Health.* (2019) 16:4515. 10.3390/ijerph16224515 31731673 PMC6888501

[39] McDonald M Currie BJ Carapetis JR. Acute rheumatic fever: a chink in the chain that links the heart to the throat? *Lancet Infect Dis.* (2004) 4:240–5. 10.1016/s1473-3099(04)00975-2 15050943

[40] Wyber R Lizama C Wade V Pearson G Carapetis J Ralph AP Improving primary prevention of acute rheumatic fever in Australia: consensus primary care priorities identified through an edelphi process. *BMJ Open.* (2022) 12:e056239. 10.1136/bmjopen-2021-056239 35273057 PMC8915338

[41] Kerrigan V Kelly A Lee AM Mungatopi V Mitchell AG Wyber R A community-based program to reduce acute rheumatic fever and rheumatic heart disease in northern Australia. *BMC Health Serv Res.* (2021) 21:1127. 10.1186/s12913-021-07159-9 34670567 PMC8527302

[42] Haynes E Mitchell A Enkel S Wyber R Bessarab D. Voices behind the statistics: a systematic literature review of the lived experience of rheumatic heart disease. *Int J Environ Res Public Health.* (2020) 17:1347. 10.3390/ijerph17041347 32093099 PMC7068492

[43] Oliver J Robertson O Zhang J Marsters BL Sika-Paotonu D Jack S Ethnically disparate disease progression and outcomes among acute rheumatic fever patients in New Zealand, 1989-2015. *Emerg Infect Dis.* (2021) 27:1893–902. 10.3201/eid2707.203045 34153221 PMC8237904

[44] Mitchell AG Diddo J James AD Guraylayla L Jinmarabynana C Carter A Using community-led development to build health communication about rheumatic heart disease in aboriginal children: a developmental evaluation. *Aust N Z J Public Health.* (2021) 45:212–9. 10.1111/1753-6405.13100 33970522

[45] Anderson A Spray J. Beyond awareness: towards a critically conscious health promotion for rheumatic fever in Aotearoa, New Zealand. *Soc Sci Med.* (2020) 247:112798. 10.1016/j.socscimed.2020.112798 32007766

[46] Bennett J Zhang J Leung W Jack S Oliver J Webb R Rising ethnic inequalities in acute rheumatic fever and rheumatic heart disease, New Zealand, 2000-2018. *Emerg Infect Dis.* (2021) 27:36–46. 10.3201/eid2701.191791 33350929 PMC7774562

[47] Gray LA D’Antoine HA Tong SYC McKinnon M Bessarab D Brown N Genome-wide analysis of genetic risk factors for rheumatic heart disease in aboriginal australians provides support for pathogenic molecular mimicry. *J Infect Dis.* (2017) 216:1460–70. 10.1093/infdis/jix497 29029143

[48] Rwebembera J Beaton AZ de Loizaga SR Rocha RTL Doreen N Ssinabulya I The global impact of rheumatic heart disease. *Curr Cardiol Rep.* (2021) 23:160. 10.1007/s11886-021-01592-2 34599389

[49] Vervoort D Swain JD Pezzella AT Kpodonu J. Cardiac surgery in low- and middle-income countries: a state-of-the-art review. *Ann Thorac Surg.* (2021) 111:1394–400. 10.1016/j.athoracsur.2020.05.181 32771467

[50] Vervoort D Meuris B Meyns B Verbrugghe P. Global cardiac surgery: access to cardiac surgical care around the world. *J Thorac Cardiovasc Surg.* (2020) 159:987–96.e6. 10.1016/j.jtcvs.2019.04.039 31128897

[51] Mirabel M Grimaldi A Freers J Jouven X Marijon E. Access to cardiac surgery in Sub-Saharan Africa. *Lancet.* (2015) 385:606. 10.1016/s0140-6736(15)60235-5 25706083

[52] Zühlke L Karthikeyan G Engel ME Rangarajan S Mackie P Cupido-Katya Mauff B Clinical outcomes in 3343 children and adults with rheumatic heart disease from 14 low- and middle-income countries: two-year follow-up of the global rheumatic heart disease registry (the Remedy study). *Circulation.* (2016) 134:1456–66. 10.1161/circulationaha.116.024769 27702773

[53] Marijon E Ou P Celermajer DS Ferreira B Mocumbi AO Jani D Prevalence of rheumatic heart disease detected by echocardiographic screening. *N Engl J Med.* (2007) 357:470–6. 10.1056/NEJMoa065085 17671255

[54] Nascimento BR Nunes MC Lopes EL Rezende VM Landay T Ribeiro AL Rheumatic heart disease echocardiographic screening: approaching practical and affordable solutions. *Heart.* (2016) 102:658–64. 10.1136/heartjnl-2015-308635 26891757

[55] Nascimento BR Nunes MCP Lima EM Sanyahumbi AE Wilson N Tilton E Outcomes of echocardiography-detected rheumatic heart disease: validating a simplified score in cohorts from different countries. *J Am Heart Assoc.* (2021) 10:e021622. 10.1161/JAHA.121.021622 34533041 PMC8649515

[56] Swain JD Sinnott C Breakey S Hasson Charles R Mody G Nyirimanzi N Ten-year clinical experience of humanitarian cardiothoracic surgery in Rwanda: building a platform for ultimate sustainability in a resource-limited setting. *J Thorac Cardiovasc Surg.* (2018) 155:2541–50. 10.1016/j.jtcvs.2017.11.106 29499865

[57] Reddy CL Peters AW Jumbam DT Caddell L Alkire BC Meara JG Innovative financing to fund surgical systems and expand surgical care in low-income and middle-income countries. *BMJ Glob Health.* (2020) 5:e002375. 10.1136/bmjgh-2020-002375 32546586 PMC7299051

