# Supplementary material for: The projections of global and regional rheumatic heart disease burden from 2020 to 2030
Source: Front Cardiovasc Med. 2022 Oct 18;9:941917. doi: 10.3389/fcvm.2022.941917 (PMC9622772; doi:10.3389/fcvm.2022.941917)
Supplement: Supplementary file 1 [file Data_Sheet_1.pdf]

Supplemental Material

Section 1: Table S1-S6

Table S1. Checklist of accurate and transparent global health estimates reporting (ie. GATHER checklist), related to STAR Methods.

Table S2. The prediction accuracy validation of Bayesian age-period-cohort model (<0.1 means acceptable).

Table S3. The cases of RHD-related burden in 1990, 2020 and 2030.

Table S4. The RHD-related ASIRs in 1990 and 2019, and its temporal trends from 1990 to 2019.

Table S5. The RHD-related ASMRs in 1990, 2020 and 2030, and its temporal trends from 1990 to 2019 and from 2020 to 2030.

Table S6. The RHD-related ASPRs of HF in 1990, 2020 and 2030, and its temporal trends from 1990 to 2019 and from 2020 to 2030.

Section 2: Figure S1-S4

Figure S1. The RHD-related disease burden at the global level, in different SDI regions and in 21 GBD regions. (A) The ASPRs in 1990, 2019, and 2030. (B) The ASIRs in 1990 and 2019. (C) The ASMRs in 1990, 2019, and 2030. (D) The ASPRs of HF in 1990, 2019, and 2030.

Figure S2. The RHD-related disease burden at the global level, in different SDI regions and in 21 GBD regions by sex. (A) The ASPRs in 1990, 2019, and 2030. (B) The ASMRs in 1990, 2019, and 2030. (C) The ASPRs of HF in 1990, 2019, and 2030.

Figure S3. The change trends of the RHD-related disease burden at the global level, in different SDI regions and in 21 GBD regions by sex. (A) The EAPCs in ASPR between 1990-2019 and 2020-2030. (B) The EAPCs in ASMR between 1990-2019 and 2020-2030. (C) The EAPCs in ASPR of HF between 1990-2019 and 2020-2030.

Figure S4. The ASPRs of RHD-related HF in 1990, 2019 and 2030 at the global level and in different SDI regions by age.

Table S1. Checklist of accurate and transparent global health estimates reporting (ie. GATHER checklist), related to STAR Methods.

GATHER Guidelines for Accurate and Transparent Health Estimates Reporting

Checklist of information that should be included in new reports of global health estimates

Item #	Checklist item	Reported on page #
**Objectives and funding**		
**1**	Define the indicator(s), populations (including age, sex, and geographic entities), and time period(s) for which estimates were made.	Page 2-4. MATERIALS AND METHODS
**2**	List the funding sources for the work.	Page 8-9. ACKNOWLEDGMENTS
**Data Inputs**		
For all data inputs from multiple sources that are synthesized as part of the study:		
**3**	Describe how the data were identified and how the data were accessed.	Page 2-3. Study Data and Definitions
**4**	Specify the inclusion and exclusion criteria. Identify all ad-hoc exclusions.	Page 2-4. MATERIALS AND METHODS
**5**	Provide information on all included data sources and their main characteristics. For each data source used, report reference information or contact name/institution, population represented, data collection method, year(s) of data collection, sex and age range, diagnostic criteria or measurement method, and sample size, as relevant.	Page 2-4. MATERIALS AND METHODS
**6**	Identify and describe any categories of input data that have potentially important biases (e.g., based on characteristics listed in item 5).	Page 6-8. DISCUSSION
For data inputs that contribute to the analysis but were not synthesized as part of the study:		
**7**	Describe and give sources for any other data inputs.	Page 2. We only used data collected from GBD. Available via online data source tools (http://ghdx.healthdata.org/gbd-results-tool).
For all data inputs:		
**8**	Provide all data inputs in a file format from which data can be efficiently extracted (e.g., a spreadsheet rather than a PDF), including all relevant meta-data listed in item 5. For any data inputs that cannot be shared because of ethical or legal reasons, such as third-party ownership, provide a contact name or the name of the institution that retains the right to the data.	Page 2. Available via online data source tools (http://ghdx.healthdata.org/gbd-results-tool). We do not have any data that cannot be shared.
**Data analysis**		

**9**	Provide a conceptual overview of the data analysis method. A diagram may be helpful.	Page 3-4. Statistical Analysis and Forecasting Model Development
**10**	Provide a detailed description of all steps of the analysis, including mathematical formulae. This description should cover, as relevant, data cleaning, data pre-processing, data adjustments and weighting of data sources, and mathematical or statistical model(s).	Page 3-4. Statistical Analysis and Forecasting Model Development
**11**	Describe how candidate models were evaluated and how the final model(s) were selected.	Page 3-4. Statistical Analysis and Forecasting Model Development
**12**	Provide the results of an evaluation of model performance, if done, as well as the results of any relevant sensitivity analysis.	Page 3-4. Statistical Analysis and Forecasting Model Development
**13**	Describe methods for calculating uncertainty of the estimates. State which sources of uncertainty were, and were not, accounted for in the uncertainty analysis.	Page 3-4. Statistical Analysis and Forecasting Model Development
**14**	State how analytic or statistical source code used to generate estimates can be accessed.	Page 3-4. Statistical Analysis and Forecasting Model Development
**Results and Discussion**		
**15**	Provide published estimates in a file format from which data can be efficiently extracted.	No published estimates were used in our analysis.
**16**	Report a quantitative measure of the uncertainty of the estimates (e.g. uncertainty intervals).	Page 4-6. RESULTS
**17**	Interpret results in light of existing evidence. If updating a previous set of estimates, describe the reasons for changes in estimates.	Page 6-8. DISCUSSION
**18**	Discuss limitations of the estimates. Include a discussion of any modelling assumptions or data limitations that affect interpretation of the estimates.	Page 6-8. DISCUSSION

This checklist should be used in conjunction with the GATHER statement and Explanation and Elaboration document, found on gather-statement.org

Table S2. The prediction accuracy validation of the Bayesian age-period-cohort model (<0.1 means acceptable).

	**Prevalence**	**Death**	**Impairment**
Global	0.005	0.029	0.007
High SDI	0.040	0.016	0.008
High-middle SDI	0.021	0.091	0.007
Middle SDI	0.005	0.020	0.014
Low-middle	0.014	0.035	0.004
Low SDI	0.005	0.007	0.001

SDI, socio-demographic index.

Table S3. The cases of RHD-related burden in 1990, 2020 and 2030.

Characteristics	Cases in 1990	Cases in 2020	Cases in 2030
	No. (95% UI)	No. (95% CI)	No. (95% CI)
**Prevalence**			
Global	23756847(18791683,29295709)	41688269(41018266,42358273)	48549858(45697036,51402680)
High SDI	840286(724963,955410)	1267502(1226391,1308614)	1556905(1387021,1726788)
High-middle SDI	3363720(2838208,3992200)	4409521(4353083,4465960)	4606852(4337966,4875738)
Middle SDI	8671201(6696174,10847101)	12669631(12498005,12841257)	13127031(12425731,13828331)
Low-middle SDI	6866722(5292949,8602523)	13006803(12755577,13258028)	15557231(14517157,16597304)
Low SDI	4000024(3058044,5086967)	10020956(9957636,10084275)	12748736(12138140,13359331)
**Death**			
Global	362160(326259,408222)	306000(298646,313354)	313688(291496,335881)
High SDI	27205(25433,28359)	24934(24335,25533)	27001(23853,30149)
High-middle SDI	67300(62547,73392)	36743(35689,37796)	37917(32219,43614)

Middle SDI	123159(109843,138933)	82140(79193,85087)	77272(70242,84302)
Low-middle SDI	105970(89332,129005)	116183(113261,119105)	127156(112885,141426)
Low SDI	38395(29963,48956)	47869(47060,48677)	50605(47393,53818)
**Impairment**			
Global	996907(766439,1288780)	2240712(2216756,2264669)	2922840(2752383,3093297)
High SDI	195446(143976,255514)	296626(288740,304512)	345557(315787,375327)
High-middle SDI	240692(179649,316406)	558772(549990,567555)	752526(694502,810550)
Middle SDI	319032(244534,411519)	801233(788868,813599)	1021352(949557,1093148)
Low-middle SDI	180061(137241,232610)	431323(427517,435129)	554166(523343,584989)
Low SDI	61358(45470,81270)	144818(143514,146122)	194133(184442,203824)

RHD, rheumatic heart disease; UI, uncertainty interval; SDI, sociodemographic index.

Table S4. The RHD-related ASIRs in 1990 and 2019, and its temporal trends from 1990 to 2019.

	1990	2019	1990–2019
**Characteristics**	**ASIR per 100, 000 (95% UI)**	**ASIR per 100, 000 (95% UI)**	**EAPC of ASIR (95% CI)**
**Global**	32.69(25.77,40.03)	37.40(28.60,46.74)	0.58(0.52,0.63)
**Sex**			
Female	35.54(28.16,43.36)	40.57(31.13,50.40)	0.63(0.57,0.68)
Male	29.81(23.42,36.73)	34.33(26.25,42.88)	0.55(0.49,0.60)
**SDI**			
High SDI	7.04(6.21,7.91)	5.86(5.33,6.40)	-0.58(-0.75,-0.40)
High-middle SDI	20.11(16.91,23.69)	17.04(13.68,20.68)	-0.40(-0.50,-0.30)
Middle SDI	31.19(24.06,38.89)	30.65(23.37,38.38)	0.06(-0.01,0.13)
Low-middle SDI	43.86(33.63,54.62)	46.91(35.70,58.71)	0.29(0.25,0.32)
Low SDI	58.30(44.27,73.70)	62.13(46.85,78.27)	0.29(0.27,0.31)
**Regions**			
High-income Asia Pacific	4.03(3.41,4.64)	2.46(2.12,2.81)	-2.00(-2.11,-1.89)
High-income North America	9.80(8.28,11.48)	9.35(8.50,10.31)	0.13(-0.14,0.41)
Western Europe	5.66(5.13,6.21)	3.90(3.48,4.34)	-1.45(-1.51,-1.38)
Australasia	5.17(4.67,5.73)	3.80(3.37,4.31)	-1.21(-1.38,-1.03)

Eastern Europe	14.26(12.78,16.00)	8.02(7.03,9.07)	-2.15(-2.26,-2.03)
Central Europe	12.18(11.15,13.21)	6.45(5.78,7.17)	-2.14(-2.28,-2.00)
Southern Latin America	27.25(21.30,33.81)	27.86(21.40,34.78)	0.09(0.07,0.12)
East Asia	29.31(22.86,36.45)	23.85(18.60,29.77)	-0.49(-0.67,-0.31)
Central Asia	38.06(29.56,47.14)	38.30(29.32,47.78)	-0.02(-0.07,0.04)
North Africa and Middle East	23.75(18.58,29.63)	25.61(19.71,31.91)	0.25(0.20,0.31)
Southeast Asia	18.50(14.89,22.47)	18.52(14.64,22.89)	0.03(-0.01,0.07)
Southern Sub-Saharan Africa	71.74(53.38,91.51)	72.77(54.23,92.50)	0.03(0.01,0.04)
Tropical Latin America	53.81(40.39,67.44)	54.01(40.56,68.35)	0.01(0.00,0.02)
Andean Latin America	47.91(36.01,60.71)	47.96(36.13,60.44)	0.00(-0.01,0.01)
Caribbean	45.69(34.42,57.31)	49.12(37.16,62.06)	0.24(0.23,0.25)
Central Latin America	22.91(17.95,28.05)	22.63(17.40,28.00)	-0.08(-0.15,-0.01)
South Asia	42.43(31.92,53.24)	42.98(32.15,54.22)	0.20(0.15,0.25)
Central Sub-Saharan Africa	80.20(60.20,102.43)	81.68(60.58,104.25)	0.02(-0.02,0.05)
Oceania	37.67(29.17,48.07)	40.72(31.80,51.77)	0.34(0.27,0.40)

Western Sub-Saharan Africa	53.74(40.60,68.00)	56.21(42.41,71.26)	0.17(0.15,0.20)
Eastern Sub-Saharan Africa	75.75(56.70,96.64)	80.98(60.90,103.24)	0.25(0.23,0.26)

RHD, rheumatic heart disease; ASIRs, age-standardized incidence rates; EAPC, estimated annual percentage change; UI, uncertainty interval; CI, confidence interval; SDI, sociodemographic index.

Table S5. The RHD-related ASMRs in 1990, 2020 and 2030, and its temporal trends from 1990 to 2019 and from 2020 to 2030.

	**1990**	**2020**	**2030**	**1990–2019**	**2020-2030**
**Characteristics**	**ASMRs per 100, 000 (95% UI)**	**ASMRs per 100, 000 (95% CI)**	**ASMRs per 100, 000 (95% CI)**	**EAPC of ASMRs (95% CI)**	**EAPC of ASMRs (95% CI)**
**Global**	8.94(8.04,10.12)	3.74(3.62,3.87)	2.98(2.27,3.69)	-2.98(-3.03,-2.94)	-2.27(-2.27,-2.26)
**Sex**					
Female	9.65(8.53,10.99)	3.95(3.81,4.10)	3.16(2.34,3.98)	-3.10(-3.17,-3.03)	-2.22(-2.23,-2.22)
Male	8.19(6.70,10.00)	3.50(3.38,3.61)	2.77(2.06,3.47)	-2.86(-2.89,-2.83)	-2.32(-2.32,-2.32)
**SDI**					
High SDI	2.62(2.45,2.73)	1.09(1.06,1.13)	0.90(0.63,1.18)	-3.00(-3.21,-2.80)	-1.92(-1.94,-1.90)
High-middle SDI	6.49(6.00,7.10)	1.83(1.75,1.91)	1.40(0.77,2.04)	-4.53(-4.68,-4.38)	-2.61(-2.65,-2.57)
Middle SDI	12.35(10.91,14.10)	3.56(3.39,3.72)	2.48(1.81,3.16)	-4.08(-4.15,-4.02)	-3.53(-3.54,-3.51)
Low-middle SDI	16.55(13.86,20.38)	8.23(7.91,8.56)	6.70(4.09,9.32)	-2.45(-2.54,-2.35)	-2.04(-2.05,-2.03)
Low SDI	14.28(10.83,18.74)	8.23(8.02,8.44)	6.50(5.00,8.00)	-1.87(-1.95,-1.78)	-2.33(-2.35,-2.30)

Regions					
High-income Asia Pacific	1.92(1.75,2.03)	0.77(0.74,0.80)	0.55(0.39,0.71)	-3.04(-3.11,-2.98)	-3.25(-3.30,-3.19)
High-income North America	2.34(2.18,2.44)	0.98(0.94,1.02)	0.91(0.50,1.31)	-3.10(-3.46,-2.74)	-0.82(-0.84,-0.81)
Western Europe	3.03(2.82,3.17)	1.42(1.36,1.47)	1.10(0.76,1.45)	-2.52(-2.66,-2.38)	-2.48(-2.52,-2.44)
Australasia	2.55(2.34,2.74)	1.13(1.04,1.22)	0.92(0.50,1.35)	-2.56(-2.80,-2.32)	-2.01(-2.02,-2.00)
Eastern Europe	4.91(4.56,5.28)	1.30(1.14,1.46)	1.17(-1.34,3.67)	-5.58(-6.10,-5.07)	-1.05(-1.07,-1.03)
Central Europe	6.11(5.90,6.30)	1.26(1.19,1.34)	0.95(0.37,1.52)	-5.72(-6.16,-5.27)	-2.85(-2.90,-2.80)
Southern Latin America	5.89(5.34,6.40)	2.11(2.00,2.22)	1.65(1.05,2.24)	-3.64(-3.76,-3.52)	-2.46(-2.50,-2.42)
East Asia	17.73(15.24,20.79)	3.87(3.66,4.09)	2.51(1.54,3.48)	-5.00(-5.13,-4.88)	-4.24(-4.31,-4.17)
Central Asia	7.27(7.00,7.56)	4.09(3.82,4.35)	3.13(0.72,5.53)	-2.51(-2.84,-2.17)	-2.64(-2.70,-2.59)
North Africa and Middle East	4.50(3.37,6.67)	1.60(1.54,1.66)	1.17(0.75,1.58)	-3.59(-3.74,-3.43)	-3.11(-3.13,-3.08)
Southeast Asia	3.67(3.07,4.30)	1.25(1.21,1.29)	0.93(0.62,1.24)	-3.73(-3.83,-3.63)	-2.89(-2.91,-2.88)
Southern Sub-Saharan Africa	3.94(3.37,4.43)	2.13(1.98,2.29)	1.63(0.64,2.62)	-2.03(-2.40,-1.66)	-2.64(-2.65,-2.63)
Tropical Latin America	2.81(2.66,2.98)	1.10(1.06,1.15)	0.79(0.54,1.04)	-3.05(-3.15,-2.94)	-3.27(-3.31,-3.23)

Andean Latin America	3.34(2.89,4.00)	1.07(1.00,1.15)	0.74(0.49,1.00)	-3.75(-3.85,-3.65)	-3.59(-3.62,-3.57)
Caribbean	4.69(3.81,5.74)	2.45(2.31,2.58)	2.18(1.48,2.88)	-2.12(-2.28,-1.95)	-1.16(-1.16,-1.16)
Central Latin America	2.86(2.72,3.00)	0.69(0.65,0.73)	0.60(0.31,0.90)	-5.16(-5.39,-4.94)	-1.29(-1.34,-1.25)
South Asia	20.67(16.92,26.43)	11.01(10.51,11.51)	9.22(4.55,13.88)	-2.28(-2.44,-2.12)	-1.76(-1.77,-1.75)
Central Sub-Saharan Africa	6.87(4.82,9.29)	3.83(3.66,4.00)	2.62(1.87,3.37)	-1.86(-2.08,-1.64)	-3.72(-3.75,-3.68)
Oceania	20.45(12.51,32.57)	15.68(14.85,16.50)	13.41(8.94,17.88)	-0.64(-0.74,-0.55)	-1.55(-1.56,-1.54)
Western Sub-Saharan Africa	6.42(4.95,8.54)	2.36(2.27,2.45)	1.76(1.21,2.32)	-3.43(-3.59,-3.27)	-2.87(-2.88,-2.87)
Eastern Sub-Saharan Africa	5.01(4.03,6.03)	2.40(2.31,2.49)	1.80(1.27,2.33)	-2.63(-2.72,-2.54)	-2.84(-2.88,-2.79)

RHD, rheumatic heart disease; ASMRs, age-standardized mortality rates; EAPC, estimated annual percentage change; UI, uncertainty interval; CI, confidence interval; SDI, sociodemographic index.

Table S6. The RHD-related ASPRs of HF in 1990, 2020 and 2030, and its temporal trends from 1990 to 2019 and from 2020 to 2030.

	1990	2020	2030	1990–2019	2020-2030
**Characteristics**	**ASPRs of HF per 100, 000 (95% UI)**	**ASPRs of HF per 100, 000 (95% CI)**	**ASPRs of HF per 100, 000 (95% CI)**	**EAPC about ASPRs of HF (95% CI)**	**EAPC about ASPRs of HF (95% CI)**
**Global**	24.49(18.89,31.19)	26.52(26.09,26.95)	26.84(22.09,31.59)	0.23(0.20,0.27)	0.11(0.10,0.12)
**Sex**					
Female	28.87(22.15,37.07)	31.81(31.26,32.35)	32.23(26.17,38.29)	0.28(0.23,0.32)	0.12(0.11,0.13)
Male	19.49(15.19,24.80)	20.70(20.38,21.02)	20.60(17.06,24.14)	0.21(0.19,0.24)	-0.05(-0.07,-0.04)
**SDI**					
High SDI	18.81(14.05,24.44)	15.86(15.33,16.40)	15.24(11.46,19.02)	-0.76(-0.84,-0.67)	-0.40(-0.43,-0.37)
High-middle SDI	22.62(17.06,29.46)	27.34(26.73,27.95)	28.82(22.72,34.93)	0.61(0.54,0.68)	0.52(0.52,0.53)
Middle SDI	27.75(21.32,35.62)	29.83(29.18,30.47)	28.93(23.15,34.71)	0.31(0.26,0.36)	-0.32(-0.33,-0.31)
Low-middle SDI	26.58(20.21,34.46)	28.69(28.27,29.11)	28.17(23.22,33.11)	0.27(0.24,0.31)	-0.19(-0.20,-0.18)

Low SDI	22.96(17.09,30.27)	24.14(23.80,24.48)	23.66(19.87,27.44)	0.25(0.22,0.27)	-0.20(-0.21,-0.20)
**Regions**					
High-income Asia Pacific	13.08(9.05,17.96)	10.73(10.31,11.15)	10.86(7.58,14.14)	-0.91(-1.01,-0.81)	0.12(0.11,0.12)
High-income North America	19.45(14.26,26.13)	18.04(17.07,19.01)	15.49(9.25,21.73)	-0.01(-0.16,0.14)	-1.52(-1.53,-1.52)
Western Europe	21.43(16.05,27.64)	17.23(16.67,17.79)	17.40(11.63,23.18)	-1.31(-1.53,-1.09)	0.10(0.10,0.10)
Australasia	28.63(22.02,35.75)	20.08(19.33,20.84)	20.68(11.73,29.63)	-1.27(-1.39,-1.16)	0.29(0.28,0.31)
Eastern Europe	8.50(5.83,12.16)	8.21(8.01,8.41)	8.02(5.87,10.18)	-0.22(-0.32,-0.11)	-0.23(-0.23,-0.22)
Central Europe	14.65(10.37,20.61)	13.71(13.32,14.09)	13.89(9.03,18.75)	-0.19(-0.30,-0.07)	0.14(0.12,0.15)
Southern Latin America	14.79(10.03,20.82)	15.06(14.64,15.48)	16.83(13.05,20.61)	-0.05(-0.10,-0.01)	1.12(1.04,1.19)
East Asia	42.59(33.36,54.08)	48.76(47.42,50.10)	49.76(36.43,63.10)	0.50(0.44,0.57)	0.20(0.19,0.21)
Central Asia	9.85(7.12,13.30)	9.04(8.77,9.32)	8.82(6.67,10.97)	-0.39(-0.49,-0.29)	-0.25(-0.26,-0.24)
North Africa and Middle East	11.65(8.50,15.68)	11.87(11.67,12.07)	12.11(9.97,14.26)	0.02(0.00,0.05)	0.20(0.20,0.21)
Southeast Asia	9.00(6.76,11.71)	9.59(9.43,9.74)	9.63(8.00,11.26)	0.23(0.18,0.27)	0.05(0.05,0.05)
Southern Sub-Saharan Africa	26.34(17.88,39.11)	23.03(22.41,23.64)	21.74(16.60,26.88)	-0.55(-0.62,-0.47)	-0.58(-0.58,-0.57)

Tropical Latin America	6.02(4.20,8.20)	6.26(6.11,6.42)	6.45(5.05,7.86)	0.03(-0.04,0.09)	0.30(0.28,0.31)
Andean Latin America	6.56(4.35,9.52)	8.71(8.40,9.01)	9.79(7.32,12.26)	0.87(0.84,0.90)	1.18(1.15,1.22)
Caribbean	8.26(6.11,11.00)	8.25(7.97,8.54)	8.34(6.31,10.38)	0.00(-0.02,0.01)	0.11(0.10,0.12)
Central Latin America	7.88(5.45,10.77)	7.51(7.32,7.69)	7.49(5.75,9.23)	-0.25(-0.30,-0.21)	-0.02(-0.03,-0.01)
South Asia	30.83(23.30,39.84)	32.38(31.93,32.83)	32.32(27.03,37.60)	0.13(0.10,0.17)	-0.02(-0.03,-0.01)
Central Sub-Saharan Africa	7.45(4.76,11.37)	7.30(7.03,7.57)	7.35(5.49,9.22)	-0.15(-0.18,-0.11)	0.08(0.07,0.08)
Oceania	36.20(27.70,46.89)	39.49(37.98,40.99)	39.99(29.78,50.21)	0.31(0.27,0.34)	0.13(0.13,0.13)
Western Sub-Saharan Africa	36.16(25.44,50.65)	38.96(38.26,39.66)	39.39(31.61,47.18)	0.38(0.30,0.46)	0.11(0.10,0.12)
Eastern Sub-Saharan Africa	2.20(1.43,3.35)	2.43(2.34,2.52)	2.47(1.83,3.11)	0.28(0.20,0.35)	0.13(0.13,0.13)

RHD, rheumatic heart disease; ASPRs, age-standardized prevalence rates; HF, heart failure; EAPC, estimated annual percentage change; UI, uncertainty interval; CI, confidence interval; SDI, sociodemographic index.

Figure S1

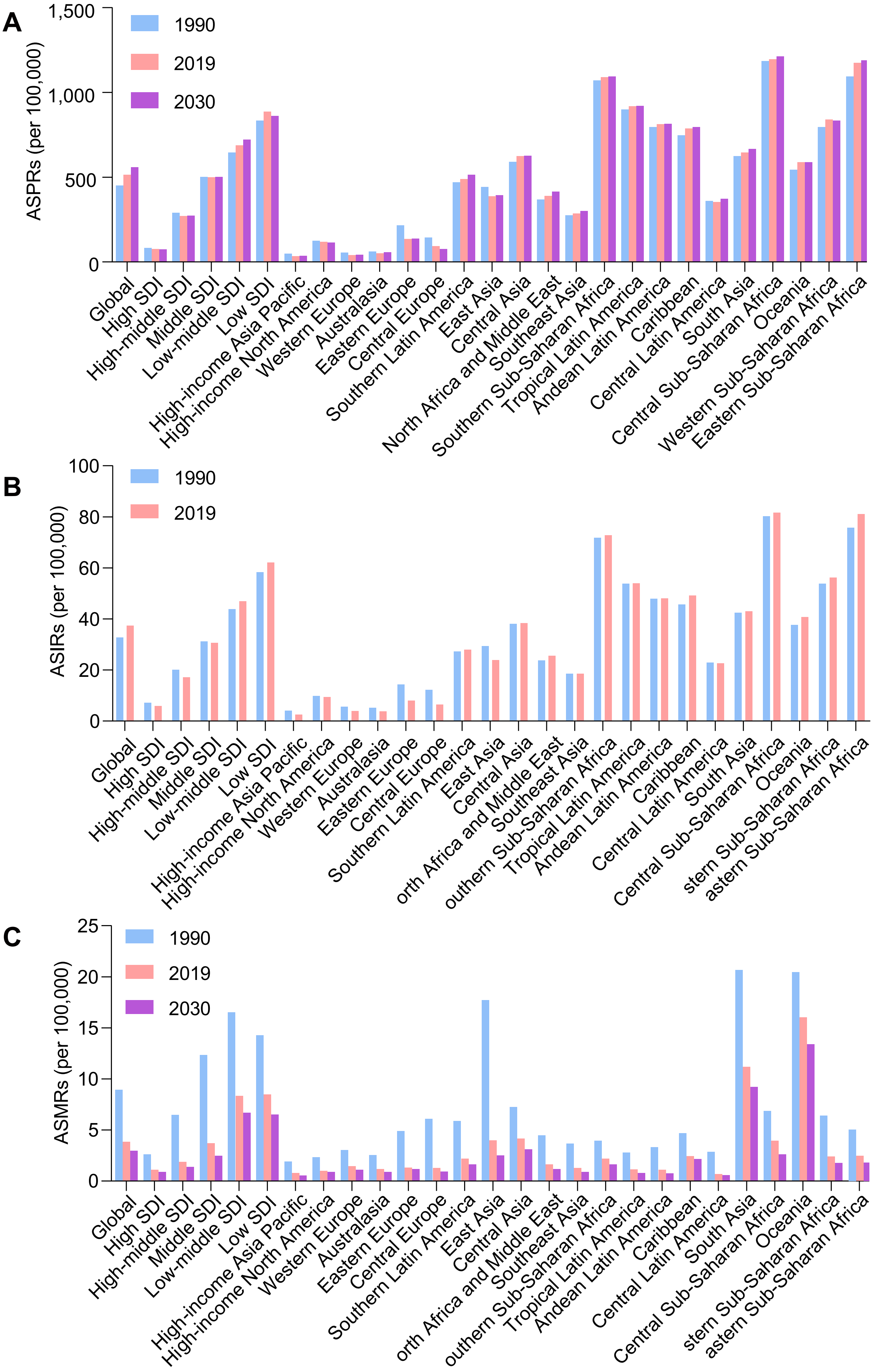
A
ASPRs (per 100,000)
0
500
1,000
1,500
1990
2019
2030
Global
High SDI
High-middle SDI
Middle SDI
Low-middle SDI
Low SDI
High-income Asia Pacific
High-income North America
Western Europe
Australasia
Eastern Europe
Central Europe
Southern Latin America
East Asia
Central Asia
North Africa and Middle East
Southeast Asia
Southern Sub-Saharan Africa
Tropical Latin America
Andean Latin America
Caribbean
Central Latin America
South Asia
Central Sub-Saharan Africa
Oceania
Western Sub-Saharan Africa
Eastern Sub-Saharan Africa
B
ASIRs (per 100,000)
0
20
40
60
80
100
1990
2019
Global
High SDI
High-middle SDI
Middle SDI
Low-middle SDI
Low SDI
High-income Asia Pacific
High-income North America
Western Europe
Australasia
Eastern Europe
Central Europe
Southern Latin America
East Asia
Central Asia
orth Africa and Middle East
Southeast Asia
outhern Sub-Saharan Africa
Tropical Latin America
Andean Latin America
Caribbean
Central Latin America
South Asia
Central Sub-Saharan Africa
Oceania
stern Sub-Saharan Africa
astern Sub-Saharan Africa
C
ASMRs (per 100,000)
0
5
10
15
20
25
1990
2019
2030
Global
High SDI
High-middle SDI
Middle SDI
Low-middle SDI
Low SDI
High-income Asia Pacific
High-income North America
Western Europe
Australasia
Eastern Europe
Central Europe
Southern Latin America
East Asia
Central Asia
orth Africa and Middle East
Southeast Asia
outhern Sub-Saharan Africa
Tropical Latin America
Andean Latin America
Caribbean
Central Latin America
South Asia
Central Sub-Saharan Africa
Oceania
stern Sub-Saharan Africa
astern Sub-Saharan Africa

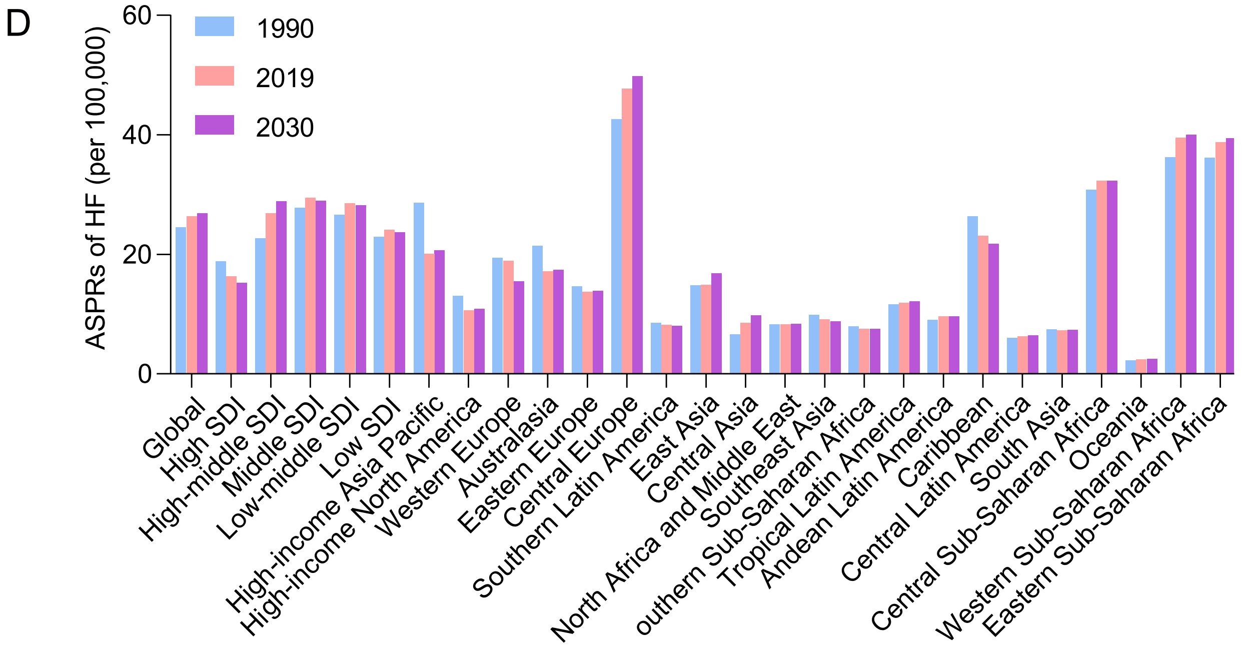

Figure S1. The RHD-related disease burden at the global level, in different SDI regions and in 21 GBD regions. (A) The ASPRs in 1990, 2019, and 2030. (B) The ASIRs in 1990 and 2019. (C) The ASMRs in 1990, 2019, and 2030. (D) The ASPRs of HF in 1990, 2019, and 2030. RHD, rheumatic heart disease; SDI, sociodemographic index; GBD, global burden of disease; ASPRs, age-standardized prevalence rates; ASIRs, age-standardized incidence rates; ASMRs, age-standardized mortality rates; HF, heart failure.

Figure S2

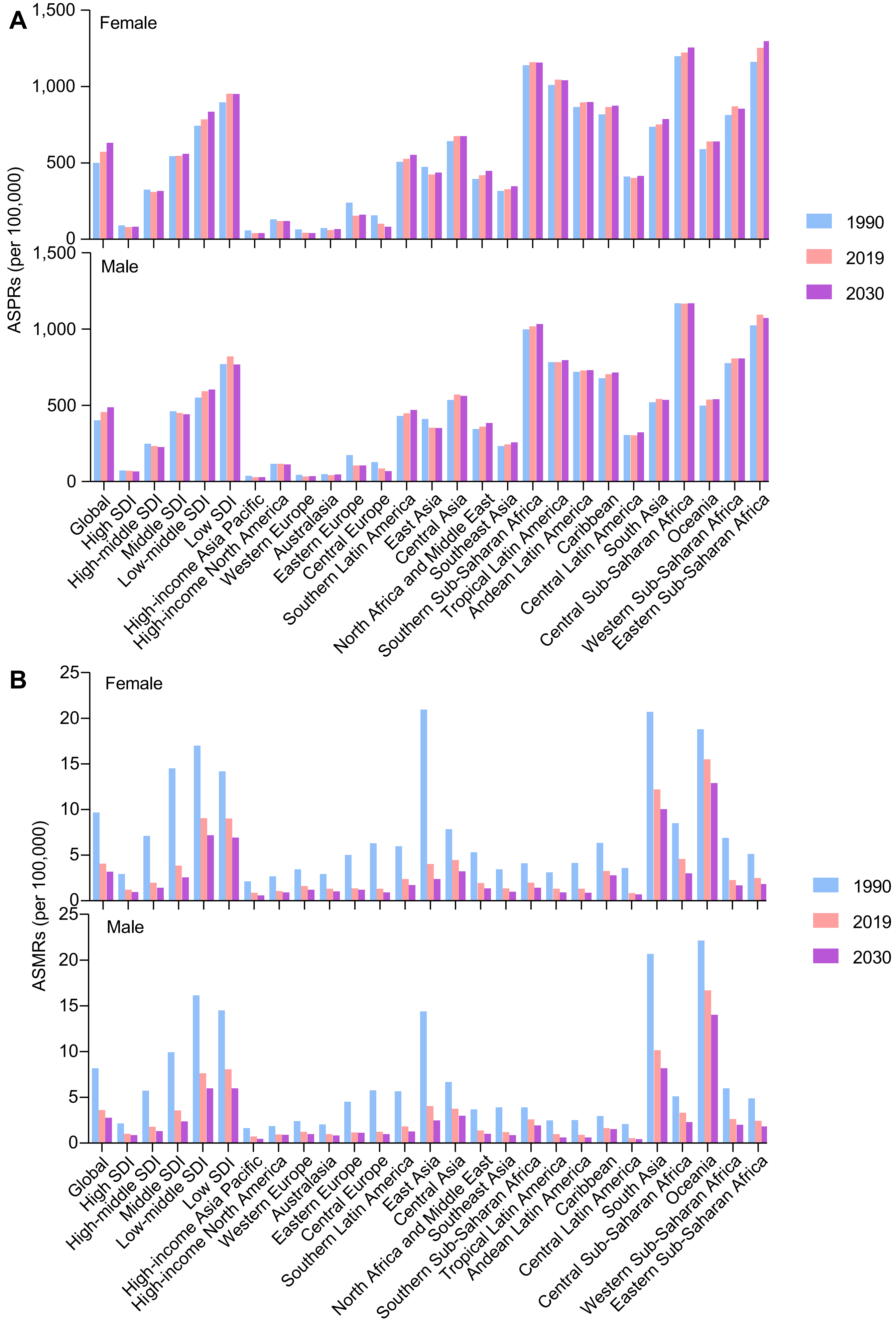
A
Female
Male
ASPRs (per 100,000)
1,500
1,000
500
0
1990
2019
2030
B
Female
Male
ASMRs (per 100,000)
25
20
15
10
5
0
Global
High SDI
High-middle SDI
Middle SDI
Low-middle SDI
Low SDI
High-income Asia Pacific
High-income North America
Western Europe
Australasia
Eastern Europe
Central Europe
Southern Latin America
East Asia
Central Asia
North Africa and Middle East
Southeast Asia
Southern Sub-Saharan Africa
Tropical Latin America
Andean Latin America
Caribbean
Central Latin America
South Asia
Central Sub-Saharan Africa
Oceania
Western Sub-Saharan Africa
Eastern Sub-Saharan Africa

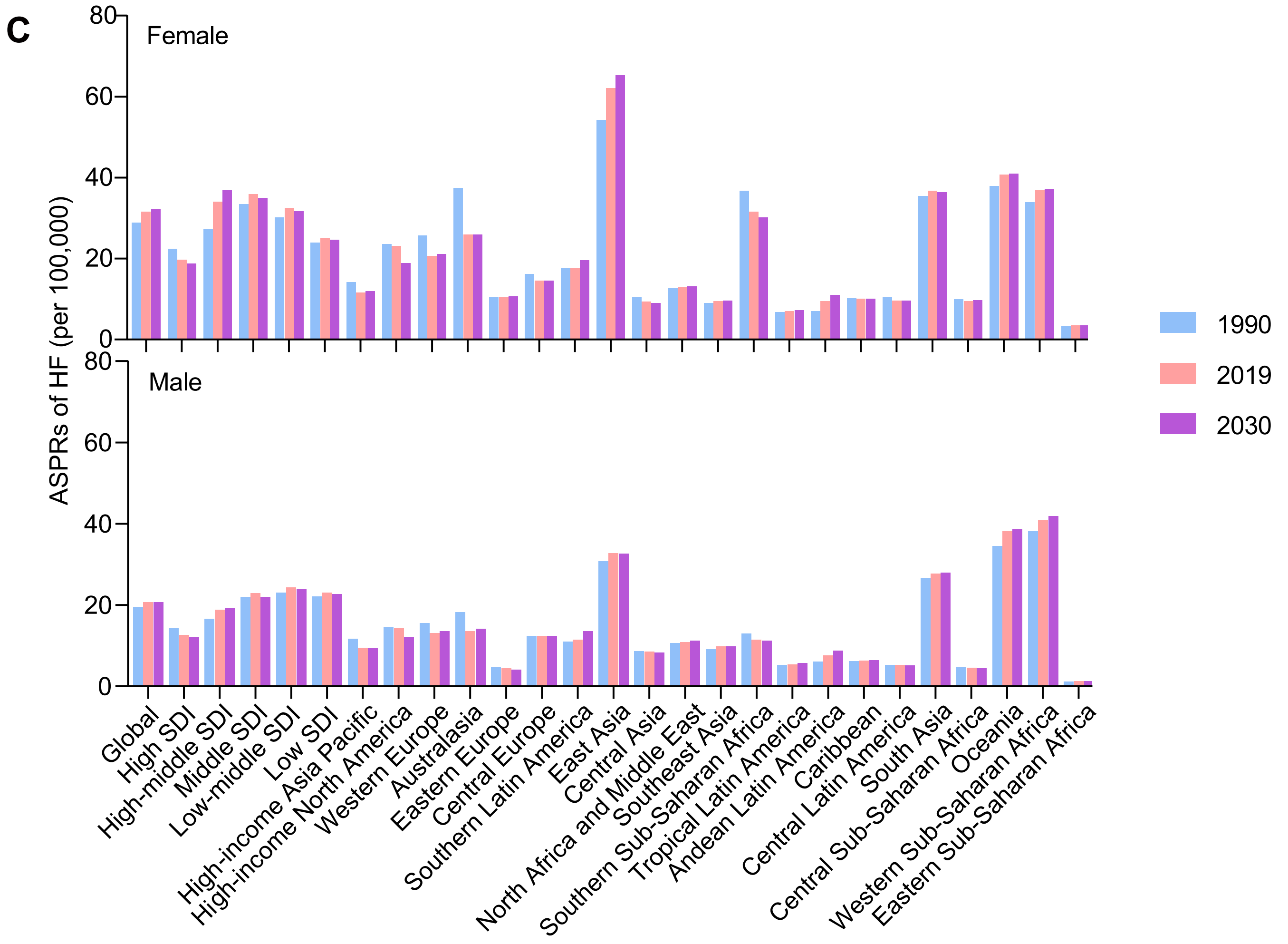

Figure S2. The RHD-related disease burden at the global level, in different SDI regions and in 21 GBD regions by sex. (A) The ASPRs in 1990, 2019, and 2030. (B) The ASMRs in 1990, 2019, and 2030. (C) The ASPRs of HF in 1990, 2019, and 2030. RHD, rheumatic heart disease; SDI, sociodemographic index; GBD, global burden of disease; ASPRs, age-standardized prevalence rates; ASMRs, age-standardized mortality rates; HF, heart failure.

Figure S3

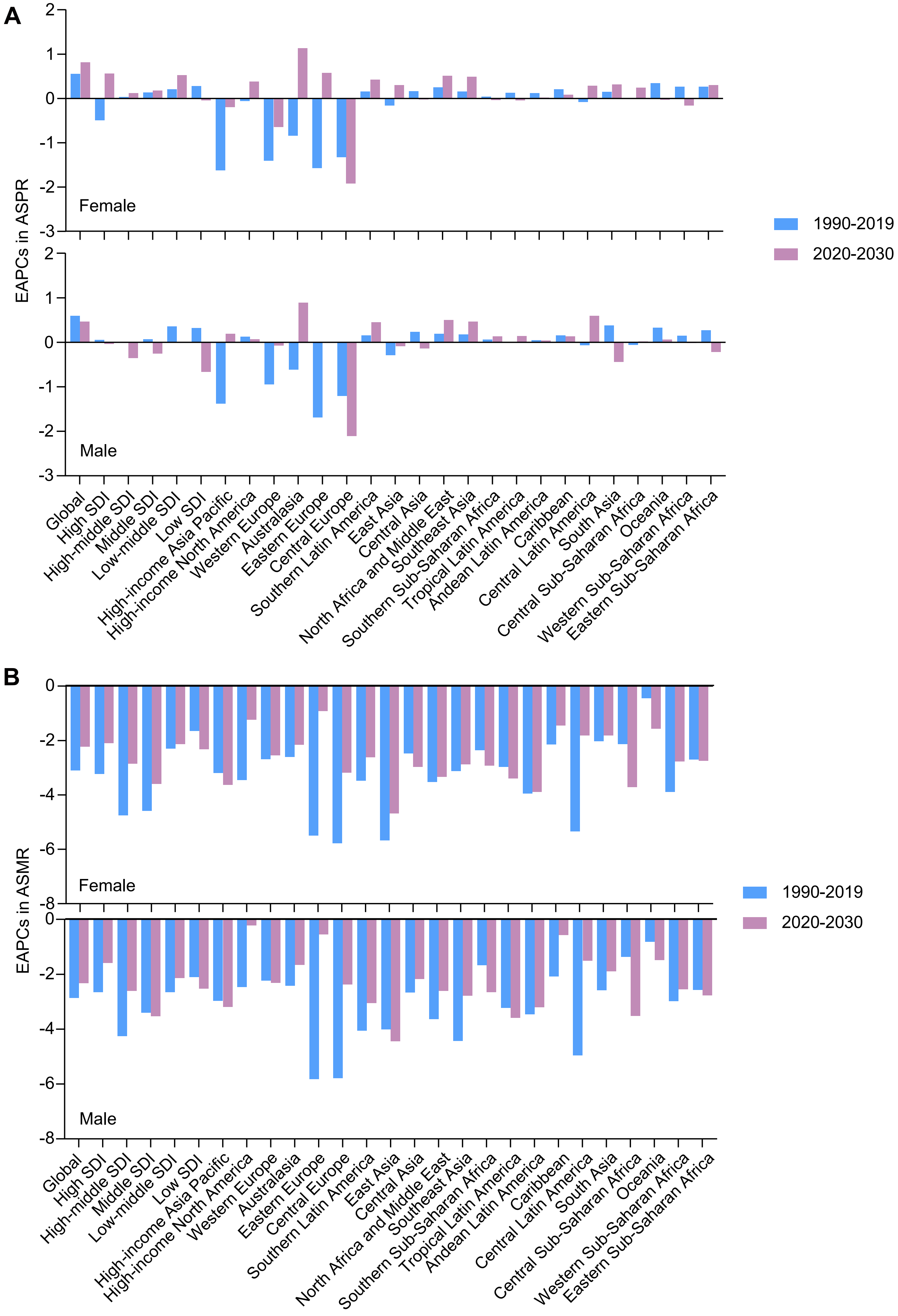
A
EAPCs in ASPR
Female
Male
1990-2019
2020-2030
2
1
0
-1
-2
-3
Global
High SDI
High-middle SDI
Middle SDI
Low-middle SDI
Low SDI
High-income Asia Pacific
High-income North America
Western Europe
Australasia
Eastern Europe
Central Europe
Southern Latin America
East Asia
Central Asia
North Africa and Middle East
Southeast Asia
Southern Sub-Saharan Africa
Tropical Latin America
Andean Latin America
Caribbean
Central Latin America
South Asia
Central Sub-Saharan Africa
Oceania
Western Sub-Saharan Africa
Eastern Sub-Saharan Africa
B
EAPCs in ASMR
Female
Male
1990-2019
2020-2030
0
-2
-4
-6
-8

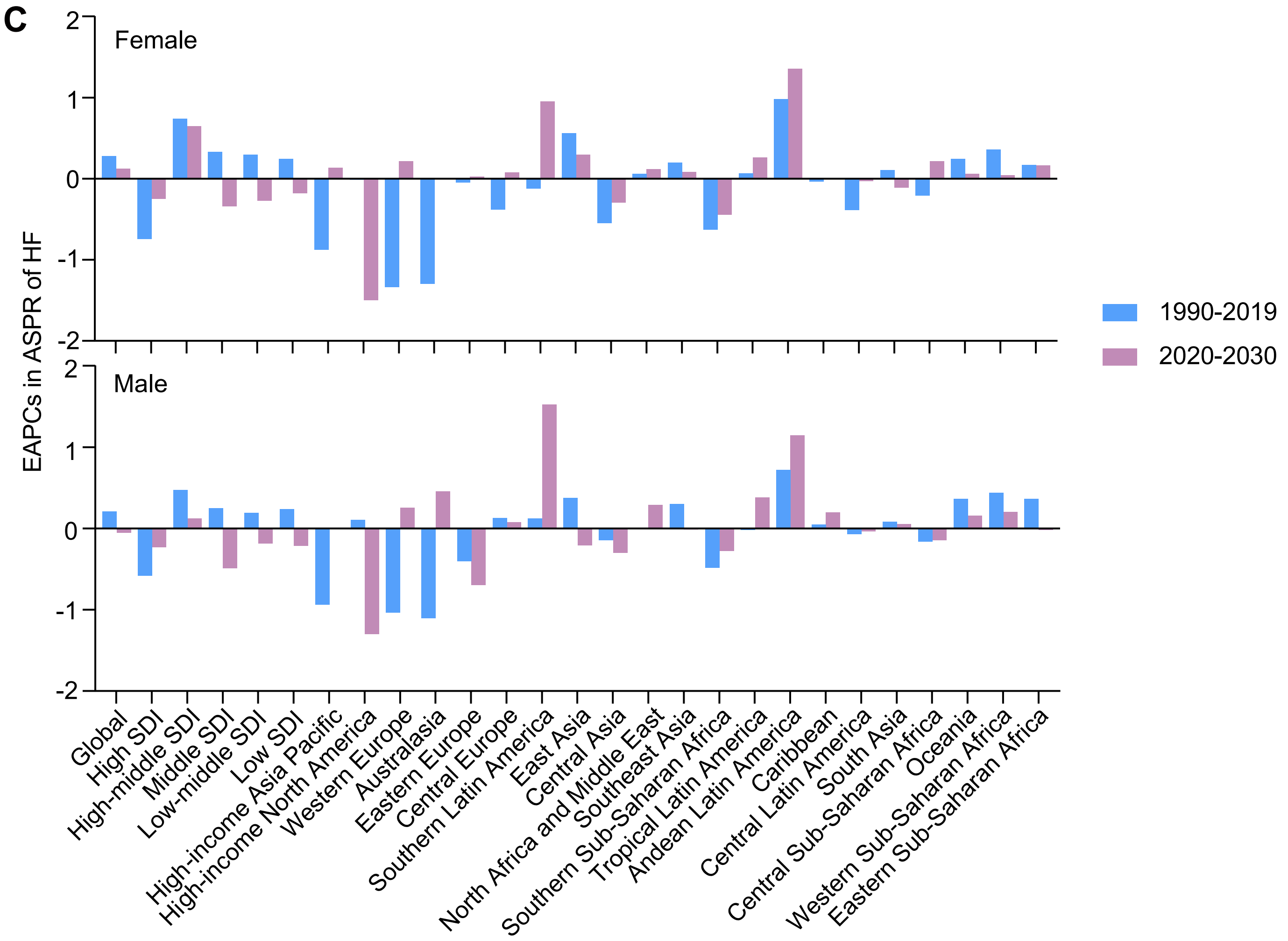

Figure S3. The change trends of the RHD-related disease burden at the global level, in different SDI regions and in 21 GBD regions by sex. (A) The EAPCs in ASPR between 1990-2019 and 2020-2030. (B) The EAPCs in ASMR between 1990-2019 and 2020-2030. (C) The EAPCs in ASPR of HF between 1990-2019 and 2020-2030. RHD, rheumatic heart disease; SDI, sociodemographic index; GBD, global burden of disease; EAPCs, estimated annual percentage changes; ASPR, age-standardized prevalence rate; ASMR, age-standardized mortality rate; HF, heart failure.

Figure S4

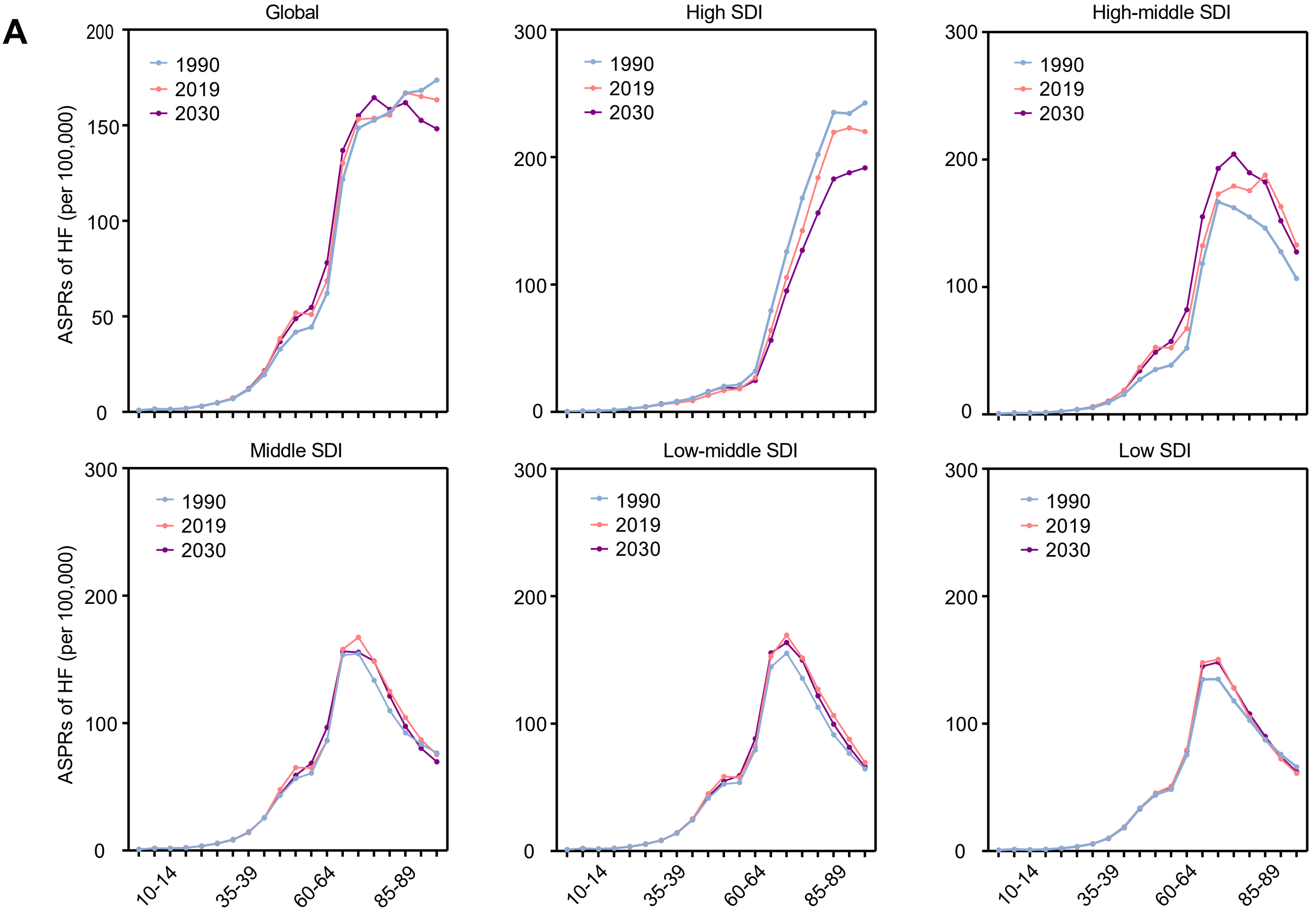

Figure S4. The ASPRs of RHD-related HF in 1990, 2019 and 2030 at the global level and in different SDI regions by age. ASPRs, age-standardized prevalence rates; RHD, rheumatic heart disease; HF, heart failure; SDI, sociodemographic index.
